# XVII Meeting of the Academy AIPMB and I National Congress SPPMB: December 2-3, 2022

**DOI:** 10.4317/medoral.1122335667799

**Published:** 2023-04-11

XVII Meeting of the Academy AIPMB and I National Congress SPPMB Abstracts of the scientific communications follow.

