# Supplementary material for: XVII Meeting of the Academy AIPMB and I National Congress SPPMB: December 2-3, 2022
Source: Med Oral Patol Oral Cir Bucal. 2023 Apr 11;28(Suppl 1):S1–S36. doi: 10.4317/medoral.1122335667799 (PMC10103586; doi:10.4317/medoral.1122335667799)
Supplement: Related texts

# Abstract: Critical update, systematic review, and meta-analysis of oral erythroplakia as an oral potentially malignant disorder

**Authors:** Y. Abouzahr, A. Lorenzo Pouso, I. Lafuente-Ibáñez, E. Otero Rey, M Pérez Sayans, A. Blanco Carrión

## Introduction

Oral erythroplakia is defined as intense red velvety plaque that cannot be characterized clinically, pathologically, or attributed to any other state. It is considered the potentially malignant disorder with the highest rate of malignant transformation to oral squamous cell carcinoma. Therefore, histopathological diagnosis and proper clinical follow-up are essential. Objectives: To evaluate the rate of malignant transformation of oral erythroplakia and identify the associated risk factors.

## Material and Methods

A literature search was performed in PubMed, Scopus, Web of Science, Embase, and LILACS.

## Results

10 observational studies with 441 patients met the inclusion requirements, with a mean malignancy rate of 12.7% and a patient follow-up of 6.66 years. At initial biopsies, 42.8% of oral erythroplakia were already oral squamous cell carcinomas. The jugal mucosa was the most frequent location of oral erythroplakia. Epithelial dysplasia was observed in the initial biopsy in all patients who had erythroplakia. The overall malignant transformation rate of the meta-analysis was 19.9%. No association was found between any clinical-pathological characteristic with the rate of malignancy.

## Conclusions

The presence of epithelial dysplasia in the initial biopsy is still considered the factor with the worst prognosis. Further observational studies with well-established diagnostic criteria and proper clinical follow-up are needed to identify the actual risk of malignancy and associated risk factors.

# Abstract: MLH1 and MGMT in oral squamous cell carcinomas and oral potentially malignant disorders

**Authors:** L. Álvarez Iglesias, E. Padín Iruegas, C. Chamorro, I. Sines Cajade, P. Madriñán Graña, A. García García

## Introduction

Methylation of the MLH1 gene appears in the early stages of oral squamous cell carcinoma (OSCC) and the MGMT biomarker in solid tumors. Objectives: To assess the levels of MHL1 and MGMT methylation in OSCC and oral potentially malignant disorders (OPMD) and to integrate this information into The Cancer Genome Atlas (TCGA) database.

## Material and Methods

Pyrosequencing analysis was performed on 85 patients to determine MLH1 and MGMT methylation: 16 patients with OSCC, 47 with OPMD who developed OSCC in the same location, and 22 with OPMD who developed OSCC in a different location. As a validation cohort, TCGA was used.

## Results

Regarding MHL1, OSCC presented the highest degree of methylation, 9.3 ± 7.3%, while MGMT, the simultaneous malignancy group, presented ten ± 13.5%, without significant differences between groups. There were also no differences in terms of sex, area, or recurrence of the lesion. Estimated survival was longer for methylated MGMT cases (19.1 months) than for unmethylated cases (9.4 months) but statistically insignificant. Study results are inconsistent with previous literature, which shows the disparity of the diseases in their histology, etiology, risk factors, etc.

## Conclusions

No relationship between MGMT and MLH1 methylation was demonstrated. MLH1 methylation was present in OSCC, while MGMT in OPMD is poorly present in cancerization, according to the TCGA database.

# Abstract: Clinicopathologic profile and evolution of lateral tongue squamous cell carcinoma in Galicia

**Authors:** I. Amezaga Fernández, C.M. Chamorro Petronacci, X. Marichalar Mendia, A. Blanco Carrión, J.M. Aguirre Urizar, A. García García

## Introduction

The lateral border of the tongue is the most frequent anatomic location for oral squamous cell carcinoma (SCC). This location has shown distinguishing clinical, pathological, and progression aspects that make lateral tongue SCC (TSCC) a particular subtype of this neoplastic disease. Objectives: To know the clinical, pathological, and evolution profile of TSCC in a cohort of patients from Galicia (Spain), diagnosed and treated in the University Hospital of Santiago de Compostela (CHUS).

## Material and Methods

In this cross-sectional study, we retrospectively reviewed all patients diagnosed and treated with TSCC in the CHUS between 2010 and 2015. We recorded the most important clinical, pathological, and progression variables. We performed a descriptive and comparative analysis of the most relevant features.

## Results

We ultimately analyzed 64 patients. The mean age was 67 years. Most were men, smokers, and drinkers and lived in rural areas. 25% presented with previous oral potentially malignant disorders (OPMD). 60,3% were diagnosed in early stages (TNM stages I and II), and 100% were histologically conventional SCCs. The 5-year overall survival was 37,5%.

## Conclusions

TSCC in Galicia shows different features compared to other regions, such as advanced age on diagnosis and remarkably high mortality. The unfavorable clinicopathologic and prognostic characteristics should be reverted in the future, implementing specific preventive measures.

# Abstract: Porphyromonas gingivalis lysate induces tissue disruption in placental explants human

**Authors:** S. Araneda Rojas, F. Fernández Moya, A. Liempi Manquel, D. Bravo Rodríguez, M. Hernández Ríos, U. Kemmerling Weis

## Introduction

Porphyromonas gingivalis (P. gingivalis) is considered a “keystone pathogen” in periodontitis and is associated with adverse pregnancy outcomes. It has been proposed that virulence factors from P. gingivalis might alter placental function. However, the effect of P. gingivalis lysates on the human placental barrier has not been studied. Objectives: To evaluate the impact of P. gingivalis lysates on the placental barrier structure.

## Material and Methods

P. gingivalis lysate were obtained by freezing-thawing- sonication. Gingipain activity and the presence of lipopolysaccharide were determined. Healthy human placental explants (HPE) obtained from term deliveries were incubated with different protein concentrations (10, 50, 100, and 200 μg/ml) of lysate for 24 hours. Conventional histochemistry and specific stains for collagen (Masson’s trichrome, Picrosirius-red) and glycosylated molecules (PAS) were used to evaluate the tissue damage. The staining intensity was analyzed using digital software (Fiji®). In addition, immunohistochemistry for laminin and fibronectin was performed (positive/ negative).

## Results

Lysate of P. gingivalis caused significant tissue damage in the free chorionic villus structure, evidenced by the separation and destruction of the trophoblast, discontinuity of basal lamina, and collagen disorganization. P. gingivalis lysates present virulence factors, such as lipopolysaccharide and gingipain protease, capable of degrading extracellular matrix components (collagen, integrin, and fibronectin), which could explain tissue destruction in HPE.

## Conclusions

The tissue alterations caused by the virulence factors of P. gingivalis in the free chorionic villus could explain, at least partially, the possible adverse pregnancy outcomes in women with periodontitis.

# Abstract: Impact of the COVID-19 pandemic on the treatment of head and neck cancer in Brazil

**Authors:** B.R. Bergo, N.P. Marques, A.R. Carlucci, D.E.C. Perez, G.D. Bressane, H. Martelli-Júnior

## Introduction

 To restrict the spread of COVID-19 infection, billions of people around the world have been quarantined or in social isolation. As a result of these health public measures, only essential services were maintained. Outpatient consultations and non-urgent surgeries were suspended to reduce hospitalizations and the risk of contagion. This context resulted in a delay in the diagnosis of several diseases, including head and neck cancer (HNC). Objectives: To assess the impact of the COVID-19 pandemic on the treatment of HNC, comparing the number of surgery and radiotherapy, and chemotherapy procedures carried out during the pre-pandemic and pandemic periods in Brazil.

## Material and Methods

We compared the mean number of HNC surgeries and the number of radiotherapy and chemotherapy procedures carried out during the pre-pandemic (from March to July 2015–2019) and pandemic period (from March to July 2020) in the five geographic regions of Brazil.

## Results

 Between 2015 and 2019, the mean number of surgeries for HNC was 5410. In 2020, during the same period, there were 3522 surgeries, representing a 35% decrease during the pandemic period. The greatest decrease was observed in the Northern region (60.7%). The number of radiotherapy and chemotherapy procedures increased by 50,8% in the pandemic period, with a higher prevalence in the Northeast (75.1%).

## Conclusions

During the peak of the COVID-19 pandemic, the data demonstrate a likely change in the HNC treatment protocol in Brazil.

# Abstract: Epidemiological trends in syphilis diagnoses during the COVID-19 pandemic in Brazil

**Authors:** B.R. Bergo, N.P. Marques, C.F. Maia, N.C.T. Marques, D.R.B. Martelli, H. Martelli-Júnior

## Introduction

Syphilis, a sexually transmitted disease caused by Treponema pallidum, is a worldwide public health problem and has an essential association with the human immunodeficiency virus (HIV) through behavioral and pathological factors. In Brazil, the syphilis situation is alarming, even with actions and strategies to control transmission implemented by the Brazilian Ministry of Health during the pandemic. Objectives: To compare the mean number of syphilis cases reported from March to December 2017–2019 with those reported in the same period of 2020 to verify if the COVID-19 pandemic is affecting the incidence of syphilis in Brazil.

## Material and Methods

A descriptive analysis and incidence rate of data extracted from the Ministry of Health of Brazil’s public database was performed.

## Results

In all Brazilian macro regions, the incidence rates of syphilis per million population increased significantly during the pandemic, reaching +78% overall, corresponding to an additional 161 incident cases per million population. The data suggest that the social distancing measures adopted during the pandemic could not interfere with general sexual behaviors in Brazil.

## Conclusions

The observed increase in syphilis cases should be further investigated to assist with decision-making processes and the programming of health measures.

# Abstract: Online survey about oral manifestations of COVID-19

**Authors:** M.G. Buccini Martínez, C.A. Aguilera Nuñez, N.R. Barreto Rodríguez, M.C. González Galván, C.M. Jara Pintos

## Introduction

Coronavirus disease 2019 (COVID-19) caused by SARS-CoV-2 (Coronavirus type 2 causing severe acute respiratory syndrome) presents systemic manifestations such as fever, anosmia, cough, sore throat and headache, dyspnea, tiredness, malaise, diarrhea. There are reports of developing dysgeusia, xerostomia, and vesiculobullous lesions as oral manifestations related to COVID-19. Objectives: To identify the most prevalent oral manifestations associated with COVID-19 in a group of Paraguayans.

## Material and Methods

 An electronic survey was used from January to March 2022. Data were presented as frequencies and percentages and analyzed using the chi2 test. Statistical analysis was performed with R software version 4.0.3.

## Results

The sample consisted of 478 people, most female from 15 to 34 years old. 65.48% stated that they had had at least one oral symptom or sign during COVID- 19. The loss in the sensation of bitter tastes (34.31%), the alteration of the taste of food (32.43%), and the loss of sweetness (32.01%) were the most prevalent symptoms. In addition, it was found that among the people who developed moderate to severe COVID-19, there was a more significant number (77.78%) of oral manifestations compared to the participants who developed it mildly (63.92%) (chi2; p= 0.044).

## Conclusions

More than half of the respondents presented oral manifestations, more frequent in those who developed moderate to severe COVID-19. 

## Results

will serve as a basis for future research and thus strengthen the surveillance of this disease.

# Abstract: Frequency of injuries in the oral region of patients admitted in the oncology service of IPS HR CDE, 2022

**Authors:** Z.A. Caballero Salinas, E.R. Montiel Fernández, E.F. Mereles Aranda

## Introduction

 The oral mucosa of patients exposed to chemotherapy and/or radiotherapy suffers lining changes, which modifies the salivary pH and the normal microbiota; consequently, injuries such as mucositis, infections, salivary gland dysfunction, sores, dysphagia, osteoradionecrosis, dental caries, and periodontal diseases are observed in these patients. They may also present with dry lips, little or thick saliva, xerostomia, and dysgeusia. Objectives: to describe the injuries in the oral region of patients admitted to the oncology service of the Instituto de Previsión Social Hospital Regional of Ciudad del Este.

## Material and Methods

Descriptive, retrospective, cross-sectional study was carried out, and were retrieved hospitalized patients’ dental records from January and February of 2022.

## Results

Data from 56% (n=23) of the patients were recorded using Microsoft Excel®, and data analysis was performed with the statistic software EPI INFO™. The confidentiality of patient data was respected, and authorization was requested from the hospital’s Teaching and Research. Most patients were from CDE, aged 21 to 79 years; the mean number of hospital days was 5.39 days (Variance 47.88, SD 6.9), and 69.57% (n= 16) were female. Coated tongue and xerostomia were prevalent in both sexes. In addition, 76.9% (n=10) had breast cancer.

## Conclusions

A significant number of oral injuries were found in hospitalized patients. The service has a dentist in the room three times a week, but the need to expand the treatment with the incorporation of the laser therapy technique is evident.

# Abstract: Whole slide imaging (WSI) technology in the diagnosis of oral pathology

**Authors:** F. Cáceres Villanueva, D. Cavagnola Espinoza, P. Ruiz Rojas, I. Espinoza Santander

## Introduction

WSI is the high-resolution digital acquisition of biological tissue sections, providing digital images that can be stored in digital data centers. WSI has been used mainly in teaching and research, having a slow introduction in the diagnostic clinic. Objectives: To validate WSI technology with highly prevalent oral pathologies.

## Material and Methods

 30 histopathological samples with prevalent diagnoses in the oral mucosa were evaluated by five dental surgeons specialized in oral pathology. After observation by conventional optical microscopy (COM) and then with WSI, the relationship between “observed diagnosis” and “true diagnosis” was determined as “diagnostic accuracy’’.

## Results

Most oral pathologists had no significant difference in diagnostic yield or time required to reach a diagnosis. Diagnostic accuracy by diagnoses was close to 100%, except for inflammatory cyst and “Other diagnoses.” Eosinophilic ulcer and neuroma stand out with lower diagnostic accuracy in the pathologies corresponding to the “Other diagnoses” group.

## Conclusions

There would not be substantial differences between WSI and COM when making histopathological diagnoses of oral pathologies because both techniques obtained high levels of precision and concordance.

# Abstract: Influence of lichenoid areas on the evolution of proliferative verrucous leukoplakia

**Authors:** L. Campo Fernández, M. Pérez Sayáns, A.I. Lorenzo Pouso, P. Gándara Vila, F. Seijas Naya, A. Blanco Carrión

## Introduction

Proliferative verrucous leukoplakia (PVL) is an uncommon oral potentially malignant disorder with a high malignant transformation rate. This disorder is characterized by its gradual topographical and histopathological changes. Objectives: To investigate the cancer incidence of PVL and its associated risk factors.

## Material and Methods

A retrospective study of 34 patients diagnosed with PVL from a university-based unit from 1995 to 2019. The follow-up was divided into four-time intervals to evaluate the clinical presentation, dysplasia grade, number of lesions, and malignant transformation rate.

## Results

The majority of patients were females, 29 (85.3%), with verrucous component (77.8%), gingival location (31.8%), and with early lichenoid areas (44.1%). The mean age of malignant transformation was 67.2 ± 12.9 years. Eleven patients (32.4%) were affected by oral cancer during the follow-up, developing 15 carcinomas. Verrucous forms presented a higher mean estimate for malignant transformation (15.2 years, 95% confidence interval of 4.4–26 years). In addition, there is an increased risk of malignancy in patients with an initial diagnosis of proliferative verrucous leukoplakia, particularly 15.55 times (95% confidence interval 1.69–143.17; p = 0.015), comparing with those who did present a preceding area with lichenoid morphology.

## Conclusions

Proliferative verrucous leukoplakia presented a high malignant transformation rate, and, sometimes, lesions with lichenoid areas are an initial stage through which PVL may start. Further studies are needed to understand the impact of these lichenoid areas in PVL.

# Abstract: Frequency of ameloblastomas, multicenter study from 2001 to 2020

**Authors:** M. Cardiel Nieves, C. Dantas Soarez, A. Alcántara Vázquez, J.C. Hernández Guerrero

## Introduction

Ameloblastoma is an aggressive benign odontogenic tumor with high recurrence, asymptomatic, slow growth, bone destruction, and soft tissue infiltration. Coming from enamel-producing cells, it makes up 11% of odontogenic tumors and 1% of maxillary tumors. It is dis covered during routine radiographic examinations. In Mexico, there are few reports of this entity. Objectives: To clarify the epidemiological findings and distribution of the histological types and patterns of Ameloblastomas.

## Material and Methods

52 cases of ameloblastomas were evaluated from 2001 to 2019. Diagnoses were determined based on WHO criteria 2017. The IBM SPSS Statistics software was used obtaining frequency, percentage, and average.

## Results

The diagnosis of ameloblastoma was confirmed in 44 cases (84.6%). The average age of patients was 33 years old, and males were predominantly affected in 53.19% of ameloblastomas. The jaw was affected in 89.36% of cases being unicystic ameloblastoma, the most frequent histologic type (50.0%), followed by conventional ameloblastomas with follicular and plexiform patterns (43.75%).

## Conclusions

Knowing the prevalence, clinical, histology, and pathogenesis of ameloblastoma will help to diagnose and prevent its evolution. However, more studies are required to know the epidemiological behavior, types distribution, and histological patterns diagnosed in the Mexican population.

# Abstract: Oral microbiome changes in patients with acquired immunodeficiency syndrome: a systematic review

**Authors:** A.R. Carlucci, B.R. Bergo, G.D. Bressane, N.P. Marques, M.K. Pugach-Gordon

## Introduction

Patients with acquired immunodeficiency syndrome (AIDS) have a considerable decrease in TCD4+ lymphocytes, which makes them more susceptible to infections by viruses, fungi, and bacteria. These infections can be severe and even lead to death. Objectives: Determine the alterations of the oral mycobiome and its clinical repercussions in HIV-infected patients, with or without treatment with antiretroviral therapy (HAART).

## Material and Methods

Electronic databases were consulted: PubMed, Scielo, BVSalud, Web of Science, Scopus, and Google Scholar, using the descriptors: “Mycobiome,” “Fungi,” “Oral,” “HIV.” Initially, 69 articles were selected, and after applying inclusion and exclusion criteria, 11 articles were analyzed and represented in this systematic review.

## Results

The literature suggests that evaluation of the salivary mycobiome in HIV infections may reflect the immunological status of the infection because the reduction of TCD4+ lymphocytes and the increase of viral load in the blood were accompanied by changes in the fungal species colonizing the oral cavity. The analyses pointed to the prevalence of species of the genera Candida, Epicoccum, and Alternaria in HIV-infected patients. In uninfected individuals, the most frequent genera were Candida, Pichia, and Fusarium, with a significant increase in Candida spp., which was related to oral candidiasis, the most common clinical oral manifestation in HIV.

## Conclusions

HAART therapy proved essential in increasing the count of TCD4+ lymphocytes and reducing the viral load in the blood, accompanied by re-establishing the balance between the oral mycobiome and the host.

# Abstract: Epidemiological and clinics characteristics of 414 cases of oral lesions and sensorial functions of COVID-19 in Brazil

**Authors:** N. Castilho, D.R.B. Martelli, R.A. Machado, C. Reis, Z.A. Cunha, H. Martelli-Júnior

## Introduction

 A cross-sectional and observational study was conducted. Approved by the Institutional Ethics Committee (#46151121.6.0000.5141). All patients were diagnosed by reverse transcription–polymerase chain reaction assay (RT-PCR) and considered to have mild symptoms, according to the latest WHO joint report. The patients were evaluated at a reference Service for COVID-19 in Minas Gerais, Brazil. The oral cavity was evaluated for each patient on the second and seventh days.

## Results

A total of 414 patients older than 18 years were evaluated. One hundred thirty-nine presented at least one of the studied conditions, oral lesions (19.08%) were the most frequently observed, followed by gustatory disorders (18.11%), xerostomia (14.25%), and olfactory dysfunction (14%). Among the oral lesions, there were various anatomical locations and clinical presentations. The occurrence involving lips and tongue represented 49 oral lesions, the most prevalent being, respectively, ulcerations (n=51), candidiasis (n=8), and erythema or red plaques (n=7). Fifty patients died.

## Conclusions

This study represents, to date, the largest case series of oral lesions in Brazilian patients with COVID-19, and oral alterations were observed in an expressive percentage of patients with COVID-19. However, it cannot be concluded that SARS-CoV-2 directly causes them.

# Abstract: Clinical and histopathological characteristics in patients with dry mouth and diagnosis of Sjögren’s syndrome, in Medellín-Colombia, 2018-2020

**Authors:** M. Correa Delgado, O. Quintero Cardona, S. Gómez Castaño, L.M. Franco González, L.V. González Pérez, S.I. Tobón Arroyave

## Introduction

Saliva is fundamental for balancing the local and general systems, such as the oral mucosa and dental structures. Its partial or total lack causes consequences that can manifest clinically and affect the patient’s quality of life. Objectives: To correlate the clinical characteristics, salivary flow, and histopathological findings in patients diagnosed with Sicca Syndrome and Sjögren’s Syndrome (SS).

## Material and Methods

A group of patients was selected with both diagnoses, sociodemographic information, salivary flow measurements, Schirmer’s test, histopathological characteristics, and classification were included.

## Results

A sample of 24 women was obtained, 14 SS negative (CH-M 0-2) and 10 SS positive (CH-M 3-4). There was a significant difference with p = 0.002 SS (-), and p = 0.019 SS (+) for unstimulated and stimulated salivary flow between the groups. In the histomorphological analysis, atrophic changes were observed in SS (-) and a higher percentage of fibrosis and fatty changes in SS (+), with a statistically significant difference for SS (+) (p = 0.047). In the association analysis, a decrease in risk was observed for those women with higher stimulated salivary flow (OR=0.37, 95% CI 0.14-0.98) and unstimulated salivary flow (OR=0.26, 95% CI 0.09- 0.78).

## Conclusions

The results of this study suggest that SS constitutes a pathology with diverse clinical presentation, which always requires a complete evaluation that includes clinical examination, measurement of salivary flow, and histopathological study to exclude other diagnoses.

# Abstract: Clinical-morphological characteristics of cutaneous and mucosal melanoma in the head and neck region: a comparative study

**Authors:** N. Cuesta Herrera, J. Rodríguez Hernández, L. Chávez Mercado, M.A. Durán Padilla

## Introduction

Head and neck melanoma constitutes approximately 25% of all melanomas and is classified according to its origin in cutaneous, ocular, or mucosal melanoma; each with specific clinical, morphological, and molecular characteristics that determine the prognosis and treatment plan. The factors associated with the unfavorable prognosis of mucosal melanoma are the difficulty of anatomical inspection that leads to late diagnosis and the surgical difficulty in removing and controlling the neoplasm. Objectives: To compare the clinical-morphological characteristics of cutaneous, ocular, and mucosal melanoma diagnosed in the Surgical Pathology Laboratory of the Hospital General de México “Dr. Eduardo Liceaga” from 2000 to 2020.

## Material and Methods

A qualitative, descriptive, retrospective study was carried out. The clinical database of all the selected slides was reviewed to obtain epidemiological information on the study population. The morphological characteristics in the slides stained with hematoxylin and eosin were evaluated with an optical microscope at 10x and 40x; pathological staging characteristics were analyzed for each anatomical region.

## Results

The most frequent melanoma was cutaneous (45%), followed by ocular (33%) and mucosal (22%); the most significant number of cases was identified in the facial region, orbit, and sinonasal mucosa, respectively. Morphologic differences by location include tumor thickness, ulceration, and the number of mitoses.

## Conclusions

In mucosal and ocular melanoma, characteristics of increased tumor thickness and a high number of mitoses predominate, probably associated with late diagnosis.

# Abstract: Quality of videos on “oral biopsy” disclosed through the youtube platform

**Authors:** A. Díaz Rodríguez, M. Diniz Freitas, J. Limeres Posse, A. García García, S. Baña Souto, A. Blanco Carrión

## Introduction

A reduced number of general dentists carry out oral biopsies during their daily praxis due to, among other reasons, the lack of training during their undergraduate studies. For dental students, YouTube has become a frequently used resource to prepare clinical procedures. However, it has been suggested that the educational quality of such videos tends to be low. Objectives: To assess the quality of YouTube videos on oral biopsy.

## Material and Methods

A search was carried out on YouTube using the term ‘oral biopsy,’ and the first 100 videos were selected. After applying the exclusion criteria, 47 videos were classified according to their duration, country, date of uploading, author, source of information, number of views, and number of likes and dislikes. The quality of the videos was analyzed according to the DISCERN and Global Quality Scale (GQS) indexes and the Video Information and Quality Index (VIQI).

## Results

Most of the videos analyzed were uploaded by dentists (78,7%), originally from India (48,9%), with an average duration of 11,8 (SD= 20,4) minutes and the number of likes and dislikes of 104,5 (SD= 186,4) and 7,1 (SD=10,55) respectively. The mean values of DISCERN, GQS and VIQI were generally low 1,3 (SD=0,52), 2,1(SD=1,04), and 9,62 (SD=1,69), respectively.

## Conclusions

Most videos published on YouTube about oral biopsies have low quality. Experts, universities, and professional organizations should try to conceal such shortages by producing videos based on scientific evidence.

# Abstract: Demographic and clinicopathological analysis of oral squamous cell carcinoma

**Authors:** P.H.S. Dourado, F.R. Pires, T.C.R.B. Santos

## Introduction

Oral squamous cell carcinoma (OSCC) is the most common malignant neoplasm of the oral cavity, affecting mainly men in the age range of 50 and 70 years. The study of this group of entities should consider the cultural habits and etiological factors most associated with a specific population, considering that the main demographic indicators and clinical information of the condition may be quite variable among countries and even in different regions in the same country. Objectives: This study aims to evaluate the demographic and clinicopathological characteristics of OSCC in a Brazilian population diagnosed at a single Oral Pathology service.

## Material and Methods

All cases diagnosed as OSCC registered in the Oral Pathology Laboratory of FO/UERJ between 2013 and 2021 were selected for the study. Clinical information was obtained from the individual laboratory records of each case. All cases’ clinical and histological information were descriptively and comparatively analyzed using the most accepted diagnostic criteria.

## Results

After the demographic analysis of the selected cases, we obtained gender data, 67% men and 33% women, with an average age of 62 years, an evolution time of 11.4 months, and a greater clinical description of an ulcerated lesion predominantly on the tongue border, 31.4% of the cases.

## Conclusions

Given this study, we obtained the clinicopathological profile of the cases of OSCC diagnosed in a Brazilian population, comparing it with the results obtained in other national and international studies.

# Abstract: Oral cancer knowledge in adults evaluated through a phone survey in the context of SARS-COV2 pandemic

**Authors:** I.L. Espinoza Santander, Y.E. Serna Arroyave, M.C. Fuentes Díaz, A. Jaramillo Rojas, K.T. Piedrahita Cortés, G.J. Alvarez Gómez

## Introduction

Oral cancer is Colombia’s 8th most common cancer, with an estimated survival rate of 52%. Lack of knowledge and awareness about oral cancer and its risk factors is associated with a delay in diagnosis. Objectives: To evaluate adults’ oral cancer knowledge through a phone survey in the context of the SARS-COV2 pandemic.

## Material and Methods

A cross-sectional questionnaire survey was conducted by phone during the COVID-19 pandemic on 268 patients attending at the dental school of Universidad de Antioquia, years 2020-2021. The collected data were analyzed using SPSS software.

## Results

The mean age was 58.9 years. 58.6% of participants had heard about oral cancer. social media was the primary source of information for 42% of participants, and 96.7% considered the screening important. Most patients did not feel worried (54.5%), fearful (59.7%), or anxious (56.3%) in case they were submitted to a screening for early detection of oral cancer. Lower socioeconomic status and educational level are correlated with lower awareness of oral cancer. The dimensions of experience and attitude towards screening were not associated with sociodemographic variables.

## Conclusions

The knowledge of oral cancer is still poor and is related to low socioeconomic and educational levels. However, this association was not observed in the attitude and experience dimensions toward screening for oral cancer. Therefore, there seems to be a need for more educational programs about knowledge of oral cancer, especially in socially disadvantaged groups, considering that there are no barriers related to screening.

# Abstract: Odontogenic cysts: clinicopathological study of a case series

**Authors:** H.K. Farias Bezerra, M.C. Assunção, K.K. Maurício Passos, J. Freire de Lisboa Castro, E.J. de Amorim Carvalho, D.E. da Cruz Perez

## Introduction

Odontogenic cysts (OC) occur in gnathic bones and consist of pathological cavities lined by odontogenic epithelium. OC are classified into developmental or inflammatory origins. Objectives: This study aimed to retrospectively analyze the clinicopathological features of all OC diagnosed in an oral pathology service.

## Material and Methods

Clinical data such as age, sex, location, clinical presentation, complaint time, diagnostic agreement, and the type of biopsy were collected from the clinical records.

## Results

A total of 517 (7.2%) cases of OC were diagnosed over 19 years. Most cases occurred in females (n = 284; 54.9%), and the mean age was 31. The mandible was the most affected site (n = 275; 39.4%), with a mean complaint time of 23 months. Most of the OC were radicular cysts (n = 148; 28.6%), followed by dentigerous cysts (n = 135; 26.1%) and odontogenic keratocysts (n = 79; 15.3%). Excisional biopsy was performed in 44.7% of cases (n = 231). In addition, the clinical hypothesis and histopathological diagnosis agreed in most cases (n = 370; 71.6%).

## Conclusions

This study found a similar prevalence of OC compared to other series worldwide. Inflammatory OC were the most frequent.

# Abstract: Qualitative research in health for the prevention and detection of early oral cancer

**Authors:** M. Farías Vergara, V. Reyes Bórquez, R. Sáez Salgado

## Introduction

According to the WHO, it is essential in health promotion to create skills in people in order to improve control over the social determinants of health. Therefore, in oral medicine, it is necessary to influence greater cultural changes, where people assume their active role as protagonists of transformations, including empowerment in the promotion of collective health and selfexamination for the recognition of oral cancer. Objectives: To build community models in oral health for the prevention and early detection of oral cancer.

## Material and Methods

Qualitative Methods, Sample: 14 leaders of social organizations in Chile, incorporated through the snowball technique. In conversation groups, in-depth interviews were conducted to define perceptions and imaginaries around oral cancer. Thematic conversation to know and build problematizations and reflections regarding the collective imaginary in oral cancer. The ideas were discussed and ordered by the same leaders in a tree of ideas according to feasibility.

## Results

Leaders propose to carry out direct activities in social spaces, beyond a dental box, for self-care of general and oral health and counseling on alcohol and tobacco consumption. Train and promote regular oral self-examination. Have training that allows local ambassadors to transfer this information and make specific educational material on learning about oral cancer and the initial oral manifestations.

## Conclusions

A survey of relevant information was achieved to carry out interventions with the community in the field of oral cancer prevention.

# Abstract: Caspase 3 and cleaved caspase 3 expression in tumorigenesis and its correlations with prognosis in head and neck cancer: a systematic review and meta-analysis

**Authors:** F. França Vieira, M.E. Padín Iruegas, M. Pérez Sayáns

 Head and neck cancer (HNC) refers to a large group of biologically similar cancers. Approximately 560.000 new cases of HNSCC and approximately 300.000 deaths are reported annually worldwide. The ability of cancer cells to evade apoptosis is one of the most important “hallmarks of cancer”. The main components of the apoptotic machinery are regulatory agents and effector agents. These regulators are part of two main circuits, the extrinsic and intrinsic apoptotic programs. Both end up generating the activation of a cascade of proteases called caspases that are synthesized as proenzymes, activated by proteolytic cleavage, and can cleave other caspases as part of the apoptotic signaling cascade. Caspase 3 is responsible for nuclear alterations in apoptosis, being considered the main effector caspase. Among these proteases, caspase 3 is the most downstream enzyme in the apoptosis-inducing protease pathway and probably the most clearly associated with cell death. The aim of this systematic review and metaanalysis was to review studies examining changes in Caspase 3 and Cleaved Caspase 3 expression in both oral premalignant disorders (OPMD) and HNC. Based on the results obtained in this study, we concluded that an increase in Caspase 3 to malignancy progression from OPMD to HNC. Possibly, a failure in the cleavage of Caspase 3 in OPMD could favor the malignancy process. The expression of Caspase 3 did not influence the prognostic values of survival in patients with HNC.

# Abstract: Immunohistochemical expression of Ki67, p53, p63, D1 Cyclin, and Bcl2 in Non-healing Chronic Traumatic Ulcers

**Authors:** G. Gilligan, R. Panico, J. Lazos, V. Palla, E. Piemonte

## Introduction

Previous studies have shown that chronic mechanical irritation (CMI) is a risk factor for Oral Squamous Cell Carcinoma (OSCC). However, there still needs to be evidence of the biological phenomenon that explains this association. Recently, a subset of non-healing traumatic ulcers (NHCTU) showed areas of early OSCC. Objectives: To characterize the immunohistochemical pattern of NHCTU located on the lateral tongue border by using biomarkers of carcinogenesis.

## Material and Methods

Descriptive, prospective study of biopsies of NHCTU Clinical and immunohistochemical variables were analyzed considering positivity according to the stained cell score and location of the Ki67-p53-p63-D1Cyclin- Bcl2 immunolabelling.

## Results

12 patients with a mean age of 67.75 years were included. 75% were female. Ki67 was positive in all the specimens, observing 41% with a high score and basal/suprabasal location. For p53, positivity was observed in 75% of the cases, but only one showed high positivity and 33% basal/suprabasal location. p63 evidenced immunostaining in all cases, with 66% of cases with intense and basal/suprabasal labeling. D1-Cyclin was positive in 66% of the cases, with 33% of the cases basal/suprabasal. Bcl2 was the biomarker with less immunostaining, with 25%.

## Conclusions

A subgroup of NHCTU shows early carcinogenic events associated with cell proliferation and cell cycle deregulation.

# Abstract: Oral microbiome and systemic antineoplastics in cancer treatment: A systematic review

**Authors:** A. González Olazabal, M. Eros Rodríguez-Fuentes, M. Pérez Sayans, P. Gándara-Vila, G. Barbeito-Castiñeiras, A. Blanco-Carrión

## Introduction

Oral mucositis is a common side effect in cancer patients receiving systemic antineoplastics. However, the underlying biological mechanisms leading to this condition are still unclear. This damage in the mucosa may be caused by an imbalance in the oral microbiota triggered by systemic antineoplastics. Objectives: Analyze changes in the different microorganisms that make up the microbiome and see if the damage caused to the oral mucosa produces imbalances.

## Material and Methods

This systematic review followed the PRISMA protocol, and the PICO question was: patients with cancer candidates for receiving systemic antineoplastics that undergo oral microbiome determinations, before and after systemic antineoplastics administration, to analyze changes in the oral microbiome composition. The bibliographic search was carried out in PubMed and other scientific repositories.

## Results

Only five articles met eligibility out of 166, just one including a control group of healthy patients. Acute myeloid leukemia (AML) was the most frequent type of cancer (40 %) among the participants. Heterogeneity in the protocols and approaches hindered a detailed comparison of the outcomes. However, it was stated that a decrease in bacteria α diversity is often associated with oral mucositis. Opposite of fungal diversity, although α diversity was lower in patients with oral candidiasis, it was not associated with oral mucositis.

## Conclusions

There is insufficient scientific evidence of oral microbiological changes in patients undergoing systemic antineoplastics. Further investigations should be carried out in patients undergoing systemic antineoplastics to identify different microorganisms developing oral mucosa damage.

# Abstract: Clinico-mycological characterization of the presence of Candida spp. in patients with peri-implantitis of the Basque Country

**Authors:** I. Lafuente Ibáñez de Mendoza, X. Marichalar Mendia, E. Eraso Barrio, G. Quindós Andres, R. Martínez-Conde Llamosas, J.M. Aguirre Urizar

## Introduction

The microbiological profile of periimplantitis (PI) is mainly bacterial; however, other agents could also be involved, such as Candida. Objectives: To determine the level of colonization of Candida spp. and its pathogenic role in PI.

## Material and Methods

Case-control study on 161 patients of the Dental Clinic Service of the UPV/EHU and the COMQ, in control and maintenance therapy of dental implants. Oral (rinse) and peri-implant fluid (paper tips) samples were cultured on CHROM/AGAR plates. Identification was made by ID32C and qPCR.

## Results

27.95% of the patients had Candida in the rinse sample. Candida spp. was isolated in 4.20% of peri-implant samples from healthy implants and in 10% of implants with PI. Most cultures were simple and corresponded to C. parapsilosis and C. albicans. Candida spp. in the oral cavity of patients with dental implants in the Basque Country is associated with edentulism, rehabilitation with overdentures and/or hybrid prostheses. The presence of Candida spp. in the peri-implant fluid is associated with an increased presence of detritus and chronic inflammation. Significant isolation of C. parapsilosis was recognized, related to the characteristics of OUR study groups.

## Conclusions

No significant differences in the presence of Candida spp. were found between patients with and without PI implants.

# Abstract: Novel variants in Brazilian patients with nonsyndromic cleft palate only

**Authors:** R.A. Machado, H. Martelli-Junior, S.R.A. Reis, E.C. Küchler, R. Scariot, L.T. das Neves, R.D. Coletta

## Introduction

Identifying genetic risk factors for nonsyndromic oral clefts is of great importance for better understanding the biological processes related to this heterogeneous and complex group of diseases. Objectives: Herein, we applied whole exome sequencing to identify potential variants related to nonsyndromic cleft palate only (NSCPO) in the multiethnic Brazilian population.

## Material and Methods

Thirty NSCPO samples and 30 sex- and genetic ancestry- matched healthy controls were pooled (3 pools with ten samples for each group) and subjected to whole exome sequencing. After filtering, the functional effects, individually and through interactions, of the selected variants and genes were assessed by bioinformatic analyses.

## Results

As a group, 399 variants in 216 genes related to palatogenesis/ cleft palate, corresponding to 6.43%, were exclusively identified in the NSCPO pools. Among those genes are 99 associated with syndromes displaying cleft palate in their clinical spectrum and 92 previously related to cleft lip-palate. The most significant biological processes and pathways overrepresented in the NSCPO- identified genes were associated with the folic acid metabolism, highlighting the interaction between LDL receptor-related protein 6 (LRP6) and 5-methyltetrahydrofolate- homocysteine methyltransferase (MTR) that interconnect two large networks.

## Conclusions

This study yields novel data on the characterization of specific variants and complex processes and pathways related to NSCPO, including many variants in the folate/ homocysteine pathway genes, and confirms that variants in genes related to syndromic cleft palate and cleft lip-palate may cause NSCPO.

# Abstract: Satisfaction of dentist users of the oral telediagnosis service in the COVID-19 pandemic, Minas Gerais, Brazil

**Authors:** K. Marinho de Paiva, L. Alves Trindade França, S. Pena Borges Pêgo, M. Mendes Lúcio da Silveira, D. Reis Barbosa Martelli, H. Martelli-Júnior

## Introduction

In early 2020, restrictions were established to control the transmission of COVID-19, such as the suspension of elective public health care. Dental teleservice has become an alternative to patient follow-up. In Brazil, the Federal Council of Dentistry published Resolution 226/2020, allowing the performance of dentistry at a distance. Objectives: To assess dentists’ satisfaction with the use of the oral telediagnosis service during the COVID-19 pandemic.

## Material and Methods

Cross-sectional and quantitative study. An online questionnaire was released to 636 dentists in the North of Minas Gerais, Brazil, between May and September 2022, with 298 respondents. The database was built using the Statistical Package for the Social Sciences®, 24.0 software. This study was approved by the CEP (#5.267.570).

## Results

There was a predominance of dentists working in the public health network (86.2%; n=257). Most professionals reported a drop in face-to-face calls during the pandemic (94.2%; n=281), and 86.2% (n=257) had losses from their activities. Teleservice resources were used during social isolation by 75.8% (n=226) of dentists, mainly for patient follow-up (63.7%; n=190). Consultation by phone was the main virtual method (63.7%; n=190). Regarding teleservice, 58.6% (n=119) of dentists realized the system was easy to use, and 50.2% (n=101) would like to continue using it after the pandemic.

## Conclusions

This is the first Brazilian study to assess dentists’ satisfaction with teleservice. It is concluded that teleservice can contribute to early diagnosis and assistance by professional users.

# Abstract: Evaluation of herpesvirus in blood and saliva of cirrhotic patients

**Authors:** G.B. Marinho, R.M. Zerbinati, N.S. Andrade, P.H. Braz da Silva, M.P. Sayáns, K.L. Ortega

## Introduction

Liver cirrhosis (LC) is closely related to immune dysfunction, caused mainly by damage to the reticuloendothelial system and hypersplenism, compromising defense cells and making the patient more prone to developing infections. In addition, human herpesviruses (HHV) are ubiquitous and latent and can be reactivated in cases of immunosuppression. Objectives: This work aims to identify HHV through PCR tests in blood and saliva samples from patients with LC to verify if the presence of these viruses is related to the population of circulating leukocytes, decompensated LC, and disease complications.

## Material and Methods

This cross-sectional observational study used blood and saliva samples from 72 patients.

## Results

The sample was predominantly male (n=51.70%), with a mean age of 54 years (SD=10). 47.2% (n=34) had leukopenia, and 59% were classified as having decompensated LC. No HHV was identified in blood samples. In saliva, the main HHV identified were HHV-7 (n=46, 63%) and EBV (n=30, 41%). The correlation between EBV with total leukocytes and with lymphocytes was statistically significant (p=0.038 and p=0.047). There was no correlation between the viral presence and other independent variables, such as LC complications or decompensated disease.

## Conclusions

In the studied population, the identification of EBV in saliva is linked to the state of circulating immunity of the cirrhotic patient. The immune deficiency presented by patients with LC may influence the shedding of EBV in saliva.

# Abstract: Association of cortisol levels and side effects of topical corticosteroids use in the treatment of atrophic/erosive omral lichen planus

**Authors:** M. Martín García, E. Blanco Aguilera, V. Sanmartín-Barragás, A. Blanco Carrión, T. García-Carnicero, P. Gándara Vila

## Introduction

It has been scientifically proven that the most effective treatment for oral atrophic/erosive lichen planus (OPLAE) is based on topical corticosteroids (TC). Despite the extensive literature, few articles study the fluctuation of basal cortisol associated with corticosteroid treatment or investigate the possible side effects of topical use of 0.5% triamcinolone acetonide (A/T 0.5%). Objectives: To describe the clinical characteristics of a group of patients with LPO-AE who will undergo topical treatment with A/T 0.5% and study the systemic effects of these TC.

## Material and Methods

This prospective observational study included 68 clinically and histologically diagnosed with LPO-AE who had to undergo treatment from June 2016 to June 2019.

## Results

This study yielded a higher prevalence of aggressive forms of LPO-AE in females than in males. Twenty percent of the patients studied had candidiasis prior to treatment. As for side effects, a partial decrease in the basal cortisol level was observed during the first phase of treatment, which was reestablished at the end of the treatment.

## Conclusions

We concluded that the use of TC for treating LPO-AE leads to a 100% improvement in symptomatology, and the appearance of side effects is linked to other local and systemic factors and the penetration capacity of the corticoid for each patient.

# Abstract: Histopathological characterization of perineural invasion in oral squamous cell carcinoma

**Authors:** R. Martínez Flores, B. Gómez Soto, C. Lozano Burgos, M. Ajudarte Lopes, W. González Arriagada

## Introduction

Oral squamous cell carcinoma (OSCC) is a malignant neurotropic neoplasm that can spread to the brainstem and peripheral nerves of the head and neck region. Some studies have attributed prognostic utility to histopathological characteristics such as perineural invasion (PNI) and nowadays is considered a minor adverse feature in treatment planning. Objectives: To describe PNI in a cohort of cases of OSCC diagnosed in a tertiary referral hospital in Valparaiso, Chile.

## Material and Methods

57 paraffin-embedded tissue samples of OSCC were included. Immunohistochemical staining for AE1/AE3 and S100 antibodies and a descriptive analysis about the location and extension of PNI and size of involved nerves were performed.

## Results

PNI was observed in 28 (49.1%) cases, and only small nerves were affected. Peritumoral PNI was the commonest location (n=17; 60,7%), followed by intratumoral PNI (11; 39,3%). There were not extratumoral PNI cases. Regarding the extent, 20 cases (71,4%) were classified as multifocal PNI, and 8 cases (28,6%) as unifocal PNI. Our findings regarding location and extent were different compared to other PNI classification-type studies. This could be explained due to the use of immunohistochemistry in our study, a recognized auxiliary technique for diagnosing PNI in OSCC.

## Conclusions

Peritumoral and multifocal PNI are frequent findings in OSCC. Immunohistochemistry for AE1/AE3 and S100 is recommended in evaluating neural invasion in the commonest malignancy of the oral cavity.

# Abstract: Lymphomas affecting the submandibular glands

**Authors:** A.L. Morais Perdigão, G. Ribeiro de Araujo, C.V. Bardález López de Cáceres, E. Roman, C. Dantas Soares, F. Paiva Fonseca

## Introduction

Lymphomas affecting major salivary glands are uncommon, and the submandibular glands are the second most affected glands. Objectives: To investigate the clinicopathological features of a series of lymphomas affecting the submandibular glands and to compare with the literature.

## Material and Methods

Lymphomas affecting the submandibular glands diagnosed from 2003 to 2019 were retrieved from two pathology files. All cases had their diagnoses confirmed according to the revised fourth edition of the WHO classification. Lymphomas that probably represented nodal extension to the submandibular glands were excluded. Demographic and clinical data were obtained from patients’ records, and the electronic search to retrieve previous reports included cases from 2008 using the PUBMED database.

## Results

Seventeen cases were retrieved, and most were classified as MALT lymphoma (52.9%). Three were DLBCL NOS (17.6%) and two cases of follicular lymphoma (11.7%). One was diagnosed as mantle cell lymphoma, one as plasmablastic lymphoma, and one as T-cell lymphoma NOS (5.8% each). Similar to the literature, females predominated (9:7), and the mean age achieved was 54.31 years old. Five were treated with surgery, and five received chemotherapy or chemotherapy with immunotherapy. Three patients died during the follow-up period, nine were disease-free, and the other five were lost.

## Conclusions

Lymphomas involving the submandibular glands are rare and more often represent B cell subtypes, especially MALT lymphoma.

# Abstract: Performance in the diagnosis of oral mucosal lesions using digital images

**Authors:** C. Morales Gómez, G. Ojeda, D. Adorno Farías, A. Maturana Ramírez, I. Espinoza Santander

## Introduction

Sending digital images of oral mucosal lesions to a geographically distant specialist in oral pathology has been proposed as a tool to contribute to the early diagnosis of oral cancer and other lesions. Studies of diagnostic performance when evaluating oral mucosa lesions through digital images are scarce. Objectives: To describe the diagnostic performance of specialists when evaluating oral mucosal lesions of a different nature through digital images.

## Material and Methods

From the National Registry of Dentists of Chile, 20 specialists in oral pathology were randomly selected and invited to review 33 cases with digital images to deliver one or a maximum of two diagnostic hypotheses. The histopathological study and/or clinical evolution were considered the gold standard. 27(82%) were reactive, and other benign lesions, and 6 (18%) had oral cancer or potentially malignant disorders (PMD).

## Results

The total proportion of correct diagnoses was 86.5%. The lesions’ accuracy was between 40% and 100%. In oral cancer and PMD, the success rate was 88.6%, and in reactive and other benign lesions, 86%.

## Conclusions

The evaluation of digital clinical images of oral mucosa lesions is a valuable tool to contribute to the diagnostic orientation of lesions of a different nature, with a range of errors that must continue to be investigated to advance in its reduction.

# Abstract: Effects of the vaper use in oral mucosa

**Authors:** C.F. Muñoz Latorre, J.P. Aitken-Saavedra

## Introduction

The use of vapers has increased in the last decade as a replacement for conventional cigarettes, given its harmful consequences. However, evidence on the effect of vaping on oral mucosa is still limited and contradictory. Objectives: Evaluate the effect of the use of vapers on the oral mucosa.

## Material and Methods

A systematic review was executed in PubMed, Web of Science, and Scielo associating MESH terms related to oral mucosal lesions (oral lesions, hyperplasia, etc.) with the use of vapers (e-cigars, vape, etc.). Duplicates and non-relevant ones were removed.

## Results

Of 440 articles found, ten were selected. Although most do not establish the advantages of vape use in relation to the conventional cigarette on the effect on the oral mucosa, three studies indicate tissue alterations, two changes in saliva, one in microflora, and three describe changes in perfusion associated with the use of the vape. The evidence indicates that there are no significant differences in the effect on oral mucosa between people who smoke vapers and people who consume conventional cigarettes. However, both in cytological and tissue studies, the presence of nicotine, increased perfusion, alterations in saliva, and tissues, as well as changes in the microflora, indicate that people who smoke vapers should be monitored as frequently as people who consume conventional cigarettes. At the same time, there is no evidence to the contrary.

## Conclusions

There are microbiological and tissue alterations in the oral mucosa associated with vapers. However, more studies must be carried out. Patients should be monitored over time.

# Abstract: Infrared thermography analysis as a noninvasive diagnostic test for burning mouth syndrome: A case-control study

**Authors:** E. Nicolás Rodríguez, F.J. Gómez García, F. Galera Molero, J.A. Ruiz Roca, M.P. López Jornet

## Introduction

Burning Mouth Syndrome (BMS) is a multifactorial chronic disease characterized by a burning sensation, mainly in the tongue, which diagnosis is made by exclusion. Thermography is an imaging technique based on acquiring and analyzing thermal data and infrared thermography (ITR) measures the radiation given off by objects. Objectives: To evaluate the use of tongue infrared thermography as a tool for the diagnosis of burning mouth syndrome.

## Material and Methods

An ITR study was carried out in patients diagnosed with BMS according to the criteria of the International Association for the Study of Pain (n = 32) and in healthy controls (n = 35). Burning sensations, dry mouth, and taste disturbances were assessed, and three temperature values were recorded for each tongue surface (dorsal, right and left laterals, and tip), along with body and environmental temperature.

## Results

A statistically significant difference was recorded in the temperature of the dorsal surface of the tongue between the BMS group and the controls (p = 0 .01). T he a rea under the curve (AUC) was 0.731 (95% CI: 0.402–0.657; p = 0.003). The sensitivity and specificity obtained were 62% and 77%, respectively.

## Conclusions

Infrared thermography seems useful as a complementary tool for diagnosing BMS, though further studies are needed in this field.

# Abstract: Association of BMP4 in the development of non-syndromic orofacial clefts in a Brazilian population

**Authors:** L.Q.R. Oliveira, H. Martelli-Junior, S.R. Reis, R. Scariot, R.A. Machado, R.D. Coletta

## Introduction

The association between polymorphisms in the bone morphogenetic protein (BMP4) gene and the development of non-syndromic cleft lip with or without cleft palate (NSCL±P) and non-syndromic cleft palate (NSCP) appears to vary between ethnicities. Objectives: To evaluate the association of the tag-SNPs rs11623717, rs17563, rs2071047, and rs2761887 in BMP4 and SNPSNP interaction with a gene from the same pathway in the susceptibility of non-syndromic oral cleft in the Brazilian population.

## Material and Methods

800 patients with NSCL±P, 274 NSCP, and 881 controls were genotyped by applying Taqman allelic discrimination assays. The ancestry of each sample was determined with a discriminant panel containing markers of the ancestry of the Brazilian population. SNP-SNP interactions between polymorphisms in BMP4 and other previously studied genes were performed by a logistic regression analysis adjusted for genomic ancestry.

## Results

No significant association between the SNPs in BMP4 and developing NSCL±P was identified, the presence of polymorphisms rs17563 and rs2761887 were associated with the development of NSCP. In the subgroup with high African ancestry, rs2761887 show significant associations with NSCL±P and NSCP. Thirteen risk SNPSNP interactions for NSCL±P involving BMP4 and FGFR1, GREM1, NOG, VAX1, and the 4p16.2 were found.

## Conclusions

Our results demonstrate an association between rs17563 and rs2761887 with NSCP and rs2761887 in patients with NSCL±P and NSCP with high African ancestry. SNP-SNP interactions between BMP4 and genes of the same pathway may also be related to the pathogenesis of this common craniofacial malformation.

# Abstract: Correlation between adverse reaction in the face and neck region and aesthetic filling materials: a systematic review

**Authors:** L.Q.R. Oliveira, H.S.C. Nicolau, H. Martelli-Junior, R.D. Coletta, R.A. Machado

## Introduction

Over the years, many materials of biological and nonbiological origin have been used to correct signs of aging on face and neck. In response, adverse reactions are seen after the procedure. Objectives: To summarize the adverse reaction in patients undergoing filling materials in face and neck.

## Material and Methods

The review was reported per the PRISMA checklist, and a literature search was conducted in databases and gray literature. We included studies published in any language mentioning adverse reactions in patients with aesthetic filling materials in face or neck. Risk of bias was assessed using the JBI appraisal tools. After a 2-step selection process, 74 studies were included: 51 case reports, 18 serial cases, and 5 cohorts.

## Results

Overall, 303 patients were assessed and lesions were observed in the lip (18%), nasolabial folds (13%), cheeks (13%), chin (10%), submental (8%), and glabella (7%). The histopathological analysis showed a foreign body granuloma (81.7%), inflammatory granuloma (3%), lipogranuloma (3%), xanthelasma-like reaction (2.3%), and other lesions less frequent. Two patients showed keratoacanthoma and two sarcoidoses after cutaneous filling. The most used materials were silicone filler (19.7%) and hyaluronic acid (15.5%). All the patients were treated, and twelve had complications.

## Conclusions

There is evidence that different fillers in specific sites can cause adverse reactions. Also, adverse reactions could be diagnosed as well as exacerbate systemic diseases. Therefore, a systemic evaluation and histopathological analysis are important for the correct diagnosis of lesions.

# Abstract: Clinicopathological aspects of odontogenic keratocyst

**Authors:** N. Otero Gayoso, B. Rivas Mundiña, M. Pérez Sayans, A. García García, L. García-Caballero Pérez, J.R. Antúnez López

## Introduction

Odontogenic keratocyst (OKC) is a pathology of the jaws that have been included in the 2017 WHO Classification of Odontogenic and Maxillofacial Bone Tumors in the group of developmental odontogenic cysts. Objectives: To describe the main sociodemographic and clinicopathological findings of a sample of OKCs from the Hospital Clínico Universitario de Santiago de Compostela.

## Material and Methods

A retrospective study was designed in which 22 OKCs were reviewed, and the patients’ sociodemographic characteristics and the lesions’ clinicopathological findings were described.

## Results

The mean age at the presentation of the OKCs was 47 years, with predilection for male patients (53%) and mandibular location (79%). Forty-one percent of patients were asymptomatic. Microscopically, they had a fibrous capsule lined by epithelial layer and presenting keratin content. Fifteen percent of the patients had some recurrence post-treatment within five years of follow-up.

## Conclusions

OKCs should be diagnosed and treated early due to their aggressive biological behavior and ability to relapse compared to other jaw cysts.

# Abstract: Teleconsultation in oral medicine during the covid-19 pandemic: experience of a Brazilian center

**Authors:** S.B. Pêgo, L.C. Farias, L.A.N. Santos, M.C.B. Barbosa, A.M.B. Paula, H. Martelli Júnior

## Introduction

Universities represent important Centers for public health assistance. However, in the context of the COVID- 19 pandemic, most Brazilian universities have suspended their academic activities and outpatient care. Objectives: Describe how the Teleconsultation Program in Oral Medicine of the School of Dentistry, Brazil, remotely contributes to counseling dentistry on diagnosing oral lesions and providing guidance on treating patients in the North Macro-region of Minas Gerais state.

## Material and Methods

Teleconsulting takes place remotely; the professional sends by smartphone, via WhatsApp®, the case report, clinical images, and complementary exams, if appropriate. Concerning this, the specialist team analyzes the clinical case and returns it to the professional sender, providing information and suggestions on the oral lesions’ diagnostic assessment and clinical management.

## Results

Since the beginning of the program, in 19 months, dentists from 40 municipalities were counseled, which resulted in 287 teleconsultations; from these cases, 103 cases were conducted face-to-face consultations in our Oral Diagnosis Service, and 38 cases were hypothesized as malignant lesions in the oral cavity and had their medical treatment conducted and followed by a multidisciplinary team, when appropriate.

## Conclusions

Teleconsultation Program represents an important tool to strengthen the communication between professionals of public health, improve health work processes, and promote better clinical guidance in Oral Medicine.

# Abstract: Analysis of CD34 expression in primordial odontogenic tumor, ameloblastic fibroma and tooth germ with artificial intelligence

**Authors:** V. Pereira Prado, F. Martins Silveira, G. Landini, A. Mosqueda Taylor, P.A. Vargas, R. Bologna Molina

## Introduction

 Primordial odontogenic tumor is a benign mixed neoplasm of recent description, which has histological similarities with other odontogenic tumors, such as the ameloblastic fibroma. In this paper, we investigate the architecture of the sub-epithelial layer of mesenchymal cells expressing the marker CD34 in primordial odontogenic tumors. Objectives: Analyze the spatial patterns of CD34 expression in primordial odontogenic tumors and compare them with those in ameloblastic fibroma and the normal tooth germ using objective imaging approaches in order to better characterize these lesions.

## Material and Methods

Two cases of primordial odontogenic tumor, four cases of ameloblastic fibroma, and two cases of tooth germ in cap and bell stages were used for morphological, structural, and immunohistochemical analyses.

## Results

CD34 expression was found in vascular endothelium of primordial odontogenic tumor, ameloblastic fibroma, and tooth germ. In addition, a characteristic mesenchymal sub-epithelial expression was observed only in primordial odontogenic tumors, corresponding to 84-86% of the sample boundaries. Moreover, the zone expressing CD34 corresponded with higher cellularity, which was absent in ameloblastic fibroma and tooth germ.

## Conclusions

Image analysis of the primordial odontogenic tumor architecture revealed characteristics absent in other odontogenic tumors and tooth germs, which supports the already existing information that this neoplasm is a distinct entity from early-stage AF or developing odontoma.

# Abstract: Expression of mitochondrial dynamic markers in adenoid cystic carcinoma

**Authors:** M.S. Pereira Sant’Ana, C.V. Bardález López De Cáeres, L. Ambrosio Lima, C. Dantas Soares, P.A. Vargas, F. Paiva Fonseca

## Introduction

Mitochondrial fission and fusion processes are known as mitochondrial dynamics, and mitochondrial activity imbalances are related to many human cancers’ pathogenesis. However, the importance of mitochondrial dynamics in malignant salivary gland tumors remains unknown. Objectives: To investigate mitochondrial dynamics prognostic significance in adenoid cystic carcinoma (AdCC).

## Material and Methods

Fifty-seven formalin-fixed paraffin-embedded cases were retrieved and disposed of in tissue microarray. Histological sections were submitted to immunohistochemical reactions against AMT, DRP1, FIS1, MFN1, MFN2, and OPA1 proteins. In addition, clinical data were retrieved from the patient’s medical files, including specific and disease-free survival data. 

## Results

It was observed that 50.9% of the cases were strongly positive for AMT and DRP1, and 49.1%, 21.1%, 22.8%, and 24.6% were strongly positive for FIS1, MFN1, MFN2, and OPA1, respectively. Reactions were observed in both the epithelial and myoepithelial components of the tumor. The higher expression of MFN2 was associated with a solid microscopic pattern (p = .016). DRP1 overexpression showed a trend towards a shorter overall survival (p = .054). In contrast, negative/weak OPA1 showed a trend towards a lower DFS (p=.051) in the univariate analysis, but no mitochondrial marker represented an independent prognostic determinant under multivariate analysis.

## Conclusions

In conclusion, mitochondrial dynamics markers seem not carry a prognostic significance for AdCC patients, but these proteins may play an important role in its pathogenesis.

# Abstract: Oral chronic hyperplastic candidiasis and its potential risk of malignant transformation: A systematic review and prevalence meta-analysis

**Authors:** A. Pérez Jardón, A.I. Lorenzo Pouso, V.C. Alberto Caponio, C.M. Chamorro Petronacci, C. Menéndez Valdés, M. Pérez-Sayáns

## Introduction

Chronic hyperplastic candidiasis (CHC) is a prototypical oral lesion caused by chronic Candida infection. A significant controversy surrounding CHC is whether this oral lesion owns malignant transformation (MT) potential. Objectives: The present study aimed to evaluate the current evidence on the MT of CHC and determine the variables that significantly influence cancer development.

## Material and Methods

Bibliographical searches included PubMed, Embase, Web of Science, Scopus, and LILACS. The cohort studies and case series used to investigate the MT of CHC were deemed suitable for inclusion. The Joanna Briggs Institute scale measured the quality of the enrolled studies. Moreover, we undertook subgroup analyses, assessed minor study effects, and conducted sensitivity analyses.

## Results

From 338 studies, nine were finally included for qualitative/ quantitative analysis. The overall MT rate for CHC across all studies was 12.1% (95% confidential interval, 4.1–19.8%). Subgroup analysis showed that the MT rate increased when the pooled analysis was restricted to poor-quality studies.

## Conclusions

According to the retrieved evidence, it remains complex to affirm whether CHC is an individual and oral, potentially malignant disorder. Prospective cohort studies to define the natural history of CHC and a consensus statement to clarify proper diagnostic criteria are strongly needed.

# Abstract: Prevalence of aphthous stomatitis in patients with inflammatory bowel disease after the treatment with monoclonal antibodies: a systematic review and meta-analysis

**Authors:** B. Pinzón, O. Salgado-Peralvo, M.D. Reboiras López, M.V. Mateos-Moreno, M. Torres-López, M.R. Garcillán-Izquierdo

## Introduction

The most frequently employed therapies in treating inflammatory bowel diseases (IBD) are monoclonal anti- TNFs and anti-integrin therapies, such as vedolizumab (VDZ). 47% of these patients present extraintestinal manifestations, the second most prevalent being aphthous stomatitis (AS). Objectives: The present study aims to investigate which of the two therapies is associated with a lower prevalence of AS after treatment.

## Material and Methods

An electronic search of the MEDLINE, Web of Science, SCOPUS, LILACS, and OpenGrey databases was carried out.

## Results

7 studies were included that met the established criteria. Six analyzed the prevalence of AS in Crohn’s disease (CD) patients, and 4 in Ulcerative Colitis (UC). A total of 1,744 patients were analyzed. The most significant reduction in AS prevalence was observed after anti-TNF therapy. The effect of these therapies on the prevalence of AS in patients with IBD-U could not be determined. The results of the meta-analyses show a trend towards statistical significance in terms of the lower prevalence of AS in patients treated with anti-TNFs compared to those treated with anti-integrins (VDZ)

## Conclusions

Both biological therapies reduce the prevalence of AS in IBD patients. However, the best results were obtained in patients treated with anti-TNFs, possibly because VDZ is often used in patients who do not respond adequately to previous treatment with anti-TNFs and because of its intestinal specificity.

# Abstract: Gingival exophytic lesions as first oral manifestation of primary lung adenocarcinomas: Systematic review

**Authors:** A. Pose Miguel, S. Piñeiro Donís, A. García García, P. Gándara Vila, P. Boñar Álvarez, A. Blanco Carrión

## Introduction

Metastases from malignant tumors in the oral cavity are rare and comprise approximately 1% of all oral neoplasms, more frequent in the jaws than in the soft tissues. The presentation of gingival metastasis as the first manifestation of a primary tumor in the oral cavity is extremely rare. Objectives: This review aims to update the diagnosis and management information of oral metastasis as the first clinical manifestation of the tumor and review all the cases published to date, providing a new original clinical case with particular histopathological characteristics.

## Material and Methods

We reviewed the literature to find all the cases of primary lung tumors that appeared with gingival metastasis as the first manifestation.

## Results

15 articles (one of them with two different cases) met the inclusion criteria. Of the 15 articles, six were rated as medium quality (40%) and nine as high quality (60%). We present a lung adenocarcinoma that first manifested as a metastasis in the gum, simulating a benign lesion.

## Conclusions

We consider the dentist’s role in the early diagnosis of oral neoplasms to be fundamental through a thorough examination of the oral mucosa. Its early diagnosis can be a determining factor in assessing the evolution and prognosis of the patient. In addition, the unusually high growth rate of this lesion should conduct to have other diagnostic possibilities, and histopathological confirmation is essential.

# Abstract: Vitamin D and its effect on patients with potentially malignant lesions and oral cancer. A systematic review

**Authors:** V. Riallin De Cruz, J.A. Ruiz Roca, F. Gómez García, F. Galera Molero, M.P. López Jornet

## Introduction

The risk of oral squamous cell cancer (OSCC) and oral potentially malignant disorders (OPMDs) has been related to low serum levels of vitamin D. Its antineoplastic activity has been demonstrated in preclinical studies in different types of malignant tumors, including OSCC, but it is necessary to know if there is clinical evidence to ensure its use in patients. Objectives: To analyze clinical studies on patients that study the effect of vitamin D or derivatives (calcitriol) in the modulation of potentially malignant lesions and/or oral cancer.

## Material and Methods

A systematic review was carried out in Pubmed, Web of Science, and Scopus, including original studies from the last ten years, on the use of vitamin D in people with OPMDs and oral cancer. Preclinical or experimental in vivo, in vitro, or non-patient studies were excluded.

## Results

Eight articles were selected after applying the inclusion and exclusion criteria. Clinical studies on these pathologies and the use of vitamin D are insufficient. Most evaluate serum levels, confirming the existence of low values of vitamin D. Improvement in quality of life, less therapeutic toxicity, and changes in the levels of immune regulatory cytokines have been observed after oral supplementation.

## Conclusions

More clinical studies are needed to clarify protocolized prescription guidelines and promote the safe use of vitamin D in these patients.

# Abstract: Lymphomas affecting the Sublingual glands: A clinicopathological study

**Authors:** G. Ribeiro de Araújo, A.L. Morais-Perdigão, J.M. Aguirre Urizar, H.A. Rebelo Pontes, C. Dantas Soares, F. Paiva Fonseca

## Introduction

Lymphomas affecting the sublingual glands are extremely rare, and few case reports are currently available, avoiding a better understanding of this clinical presentation. Objectives: The current study aims to describe the clinicopathological and microscopic features of a series of lymphomas involving the sublingual glands.

## Material and Methods

All cases diagnosed in four pathology services were assessed, and the formalin-fixed paraffin-embedded tissue blocks were retrieved for diagnosis confirmation. Clinical data were obtained from the patient’s medical files.

## Results

We obtained seven cases of lymphomas in the sublingual glands representing two follicular lymphomas, two diffuse large B cell lymphomas not otherwise specified (DLBCL NOS), two MALT lymphomas, and one mantle cell lymphoma. In all cases, the tumor cells infiltrated the glandular parenchymal, although in two of them, the neoplastic cells were located more superficially and permeated the glandular acini and ducts. Clinically, the tumors presented as asymptomatic nodules, and one patient (affected by DLBCL NOS) died, while the other cases remained alive at the last follow-up.

## Conclusions

Lymphomas affecting the sublingual glands are usually of the mature B cell lineage, usually representing lowgrade subtypes, and may clinically resemble other more common lesions in the floor of the mouth, like salivary gland tumors.

# Abstract: Evaluation of the effect of Streptococcus dentisani on Candida albicans growth. In vitro study

**Authors:** J.G. Sánchez Medina, M. Comas García, R.E. Martínez Martínez, J.L. Cuellar Camacho, S. Aranda Romo

## Introduction

Antifungal agents as oral candidiasis treatment favor resistance. Probiotics are therapeutic alternatives: based on ecological balance. Different studies have evaluated the effect of probiotics on Candida albicans, but to date, probiotic strains are of gastrointestinal origin. Streptococcus dentisani is a promising nichespecific probiotic, its effect has only been described on bacteria. Objectives: To determine the effect of S. dentisani on the different developmental stages of C. albicans ATCC and a clinical isolate of sub-prostatic stomatitis.

## Material and Methods

Growth quantification and ultrastructural analysis with atomic force microscopy (AFM) were performed on biofilms of C. albicans ATCC 90028 and a clinical isolate. They were treated with cell suspensions and supernatants of S. dentisani CECT 7746. Cell viability was determined by quantification of colony-forming units (CFU) and an XTT reduction assay.

## Results

S. dentisani significantly (p < 0.05) reduced the CFU of Candida albicans A TCC and clinical isolate (40 - 21 %). There was greater inhibition in the clinical isolate. Supernatants significantly inhibited development, but the inhibitory effect was less than the direct effect. Microscopic analysis revealed that S. dentisani reduced the density of C. albicans in a mixed biofilm.

## Conclusions

S. dentisani has an antifungal effect on the development of C. albicans, indicating that the fungus-bacteria interaction affected the membrane integrity of the yeast. For the first time, the antifungal effect of S. dentisani on the development of C. albicans is elucidated, laying the foundation for future studies.

# Abstract: Histopatological changes induced by highpower laser surgery in oral epithelium: a systematic review

**Authors:** G.L. Santos, M.P. Mamani, D.T. Oliveira

## Introduction

High-power lasers have become important for oral soft tissue lesions treatment. One advantage of high-power lasers used in oral lesion surgery compared to the cold scalpel technique is the coagulative effect and low risk of local infection. However, the disadvantages of laser therapy include thermal damage and being unsuitable for all oral soft tissue lesions. Objectives: To describe the microscopic artifacts caused in oral mucosa induced by the high-power laser when compared to the cold scalpel.

## Material and Methods

This systematic review critically evaluated the use of a high-power laser compared to a traditional scalpel in oral tissue lesions. A comprehensive search was performed on Pubmed, Scopus, Web of Science, and LIVIVO databases. The gray literature included Google Scholar, Proquest, and Open gray.

## Results

723 articles were retrieved, and after the different stages of critical evaluation, they were reduced to 7 articles that fully met the inclusion criteria. The most observed changes were the tissue damage clefts, blisters, hyperchromic nuclei, and thermal laser damage. The cytological artifacts generated by laser in oral epithelium such as hyperchromic nuclei, intracellular vacuolization, loss of cell attachment from the lamina propria could be critical when dysplastic changes or cellular invasion are evaluated to establish an accurate diagnosis.

## Conclusions

A laser is an effective tool. However, it can induce thermal epithelial and cytological artifacts that must be considered to avoid misdiagnosis in oral biopsies and consequent errors in the treatment of the patient´s lesion.

# Abstract: Anxiety, depression, and stress in oral cleft childrens’ parents: Case-control comparative study

**Authors:** S. Trezena Costa, F.E. Soares Oliveira, C.E. Marques, N.C. Teixeira Marques, D.R. Barbosa Martelli, H. Martelli-Junior

## Introduction

The birth of a child with oral cleft directly impacts their parent’s mental health. Objectives: To compare levels of anxiety, depression, and stress in oral cleft children’s parents with clinically healthy children’s parents.

## Material and Methods

Case-control cross-sectional study with a convenience sample. The case group consisted of parents of children undergoing treatment at a Reference Center for Craniofacial Deformities – Centro Pró-Sorriso (Minas Gerais, Brazil). The Control group was made up of parents from the same region who accompanied their children for dental care at the university where the Center is located. A validated questionnaire reported anxiety, depression, and stress levels. The Research Ethics Committee approved the study under number 4,927,752.

## Results

157 parents participated (28.0% of the case group and 72.0% of the control group). The majority were women (91.1%), with a mean age of 35.6 years, and with a partner (59.9%). Moderate to severe anxiety levels were present in 20.5% in the case group and 28.3% in the control group; depression in 34.1% in the case and 29.2% in control and; stress in 38.6% (case) and 30.1% (control). There were no associations in terms of levels between groups (p>0.05).

## Conclusions

There are no differences between the levels of stress, depression, and stress in the studied’ groups. A slight predilection for depression and stress at moderate to severe levels was observed in oral clefts children’s parents.

# Abstract: Nestin expression is associated with relapses in head and neck lesions

**Authors:** S. Valencia Martínez, J.M. Somoza Martin, C. Chamorro Petronacci, F. Baltazar, M. Pérez Sayans, A. García García

## Introduction

Nestin is a protein marker of immature/stem cell-like tissue that acts as a tumoral and metastasis development effector. Objectives: To investigate the clinical significance of nestin immunohistochemical expression in head and neck lesions in patient survival and recurrence.

## Material and Methods

39 (44.3%) nasosinusal, 37 (42%) major salivary glands, and 12 (13.6%) oral cavity lesions were included.

## Results

The expression was categorized into grades, negative for 55 (62.5%) cases, grade 1 in 10 cases (11.4%), grade 2 in 12 cases (13.6%), and grade 3 in 11 cases (12.5%); 100% of pleomorphic adenomas were positive with grade 3 intensity, 100% of polyps and inverted papillomas were negative (p < 0.001). The lowest estimate of disease-free-survival (DFS) was for grade 1 expression, with 50 months, confidence interval (CI): 95% 13.3– 23.9 months, and the highest for grade 3 expression, 167.9 months (CI: 95% 32.1–105 months; Log-Rank = 14.846, p = 0.002). ROC (receiver operating characteristic) curves revealed that the positivity for nestin (+/−) to malignancy presented a sensitivity of 50.98%, a specificity of 81.08%, with an area under the curve of 0.667 (p = 0.009).

## Conclusions

Nestin could be a valuable marker to detect the presence of stem cells in head and neck tumors that have a role in tumor initiation and progression.

# Abstract: Root canal microbiota as an augmented reservoir of antimicrobial resistance genes in type 2 Diabetes Mellitus patients

**Authors:** V.R. Vázquez Ramos, R.M. Pérez Serrano, P. García Solís, R.A. Domínguez Pérez

## Introduction

Antimicrobial resistance is a global public health problem. Root canal microbiota associated with apical periodontitis (AP) represents a well-known reservoir of antimicrobial resistance genes (ARG). However, the effect of Type 2 diabetes mellitus (T2DM) in this reservoir is unknown. Objectives: To establish if root canal microbiota associated with AP in T2DM patients is an augmented reservoir by identifying the prevalence of nine common ARGs and comparing it with the prevalence in nondiabetic patients.

## Material and Methods

This cross-sectional study included two groups: A T2DM group conformed of 20 patients with at least ten years of living with T2DM and a control group of 30 nondiabetic participants. Premolar or molar teeth with pulp necrosis and AP were included. A sample was collected from each canal before endodontic treatment. DNA was extracted, and ARG was identified by PCR. 

## Results

tetW and tetM genes were the most frequent (93.3 and 91.6%, respectively), while the ermA was the less frequent (8.3%). The distribution of the ARG was similar in both groups, but a significant difference (p<0.005) was present in ermB, ermC, cfxA, and tetQ genes, being more frequent in the T2DM group. Eighty percent of the T2DM patients presented a minimum of four ARG, while 76.6% of the control group presented a maximum of three.

## Conclusions

Root canal microbiota associated with AP in T2DM patients carries a higher prevalence of ARG; hence this pathological niche could be considered an augmented reservoir.

# Abstract: Teripadatide as coadyuvant in the treatment of medication related osteonecrosis of the jaw (MRONJ)

**Authors:** P. Vázquez Calvache, A. Blanco Carrion, A. Gutiérrez Corrales, A. García García, J.M. Somoza Martín, J.L. Gutiérrez Pérez

## Introduction

Drug-induced osteonecrosis of the jaw is a complex pathology that can be associated with multiple morbidities. In this review, we will focus on drug-induced jaw osteonecrosis (MRONJ), mainly derived from antiresorptive therapies based on the parenteral administration of bisphosphonates and/or Denosumab. There are many possible treatments for this pathology, although new possibilities continue to open up, such as Teriparatide. Objectives: Our review aims to know the capacity of “Teriparatide” to act jointly as an adjuvant in treating drug-induced osteonecrosis of the jaw.

## Material and Methods

A systematic review of the literature was carried out. We included the most scientifically relevant studies published in the last five years in the databases “Pubmed” and “Scopus”. These studies studied the capacity of “Teriparatide” to act as a coadjuvant in treating MRONJ.

## Results

It was observed that several studies determined the capacity, in the absence of studies with more scientific evidence, of Teriparatide as a coadjuvant in the conservative treatment of MRONJ.

## Conclusions

It is necessary to carry out studies of more significant evidence and, with long-term evolution, to determine that Teriparatide could be considered the drug of choice in the conservative treatment of MRONJ. However, in the publications, to date, the results are promising.

# Abstract: Vaporization of the oral leukoplakia with CO2 Laser

**Authors:** G.C. Vianna Camolesi, M. Vilar Villanueva, M. Gallas Torreira, M. Pérez Sayans, A. Blanco Carrión, P. Gándara Vila

## Introduction

Oral leukoplakia (LO) is one of the most common potentially malignant disorders of the oral cavity. The prevalence is 4.11%, and the rate of malignant transformation is 9.8%. Objectives: The study’s objective was to evaluate the response to the treatment of LO with CO2 laser vaporization and identify the recurrence rate of lesions.

## Material and Methods

A retrospective study included 58 patients and 78 lesions with a confirmed diagnosis of LO and treated with a CO2 laser at the USC School of Dentistry. Recurrence was considered to be a lesion reappearing at the lasertreated site. SPSS version 20.0 (SPSS Inc) was used for data analysis.

## Results

Of the patients in the sample, 70.7% presented multiple lesions, and 29.3% had single lesions. The most frequent location was the oral mucosa (19.2%), followed by the gingival mucosa (16.7%). The mean follow-up time was 57.5 months. Discussion: The complete resolution reported rate was 47.4%, and the recurrence rate was 52.6%. Similar values with those reported in the literature.

## Conclusions

Vaporization of the LO should be performed with a safety margin of at least 3 mm to the clinical limits of the lesion. This is a key factor in the prevention of recurrence. In addition, patients must have long-term follow-ups.

# Abstract: Lip alterations in a sample of farming workers in the eastern part of Antioquia and its related factors

**Authors:** V.Y. Vong Montoya, A. Acosta Correa, Y.N. Ceballos Castro, L. López Velilla, S. Rendón Rojo, L.V. González Pérez

## Introduction

Workers in the agricultural sector are exposed to factors such as ultraviolet radiation that make them susceptible to developing lip changes, some harmless and others with the potential for malignancy. Therefore, considering Eastern Antioquia is an important agricultural region in Colombia, we proposed this study. Objectives: To determine the frequency of lip alterations in a working population sample and its related factors.

## Material and Methods

A cross-sectional study was conducted through a survey including sociodemographic, labor and habit aspects, clinical examinations, photographic records, and educational material for each participant. Fortytwo workers (76% women) participated, aged 33 (±11) years, with a mean of 36 (±53) months working in the trade and 90% trained for their functions. 100% were affiliated with social security; two-thirds consumed alcohol, 10% smoked, and 19% with the habit of nibbling objects. 62% reported cheilophagia and 31% lip suction. 79% spend six or more hours in the sun, a third of them did not use sunscreen, and >70% did not use lip balm.

## Results

Clinically, tissue losses were observed in 54.8% with statistically significant differences by sex and object nibbling habit (p<0,05) 47% had color changes with statistically significant differences according to cheilophagia habit p<0,05; lip suction p<0,01; the type of contract p<0,05. In 10%, alterations requiring follow-up were found.

## Conclusions

Lip alterations in workers in the agricultural sector are related to environmental conditions and intrinsic factors, which require strategies for epidemiological surveillance.

# Abstract: Odontoameloblastoma. Clinical case report

**Authors:** V.E. Palacios Inostroza, C.P. Caamaño Martínez, F. Merchán

## Introduction

Odontoameloblastoma (OA) is a rare mixed odontogenic neoplasm characterized by the simultaneous appearance of ameloblastoma and a compound or complex odontoma in the same tumor mass. The term odontoameloblastoma was included in the 1971 WHO classification and is defined as “a neoplasm that includes odontogenic ectomesenchyme in addition to odontogenic epithelium that resembles an ameloblastoma in both structure and behavior.” It is characterized by slow and progressive growth, similar to that of ameloblastoma.

## Case Report

We present the case of a female patient with a panoramic radiograph finding that showed a unilocular radiolucent area in the posterior mandibular, associated with a radiopaque mass in relation to a tooth in coronary formation. The histopathological study surprised with the diagnosis of odontoameloblastoma.

## Conclusions

Odontoameloblastoma is usually asymptomatic and can occur in both the maxilla and the mandible. The clinical and radiographic differential diagnoses include welldefined unilocular or multilocular radiolucent odontogenic and non-odontogenic lesions containing variable amounts of radiopaque material.

# Abstract: Oral ulceration with bone sequestration. Description of 8 cases

**Authors:** J. Alberdi Navarro, D.A. Cortés Ramírez

## Introduction

 Oral ulceration with bone sequestration (OUBS) is characterized by a localized ulcerated lesion presenting a small underlying bone sequestration in the absence of any factor associated with osteonecrosis. There is scarce information about this pathology in the literature.

## Case Report

Eight cases diagnosed with OUBS are included, corresponding to 6 men and two women, with a mean age of 55.8 years (43-76). Seven lesions are located in the posterior lingual area and one in the hard palate. The mean size of the sequestrum extracted was 0.52 cm (0.4-0.7), with a mean exposure time of 4 weeks (2-8). Three cases had previous dental treatment in the area. In all cases, the indicated treatment was the removal of the sequestrum. Unfortunately, one of the cases presented a recurrence. The presence of necrotic bone tissue with irregular edges and the presence of bacterial colonies characterized the histopathological study.

## Conclusions

Although oral ulceration with bone sequestration is a poorly described pathology, it presents a characteristic clinical expression affecting the posterior lingual mandibular area. We should think of this entity when patients present a circumscribed area of bone exposure in the absence of etiopathogenic factors associated with osteonecrosis in this location. Its correct diagnosis is essential since it presents a differentiated prognosis from other pathologies that occur with exposure to necrotic tissue.

# Abstract: Uncommon manifestation of actinomycosis in the periapical region: a case report

**Authors:** E. Almeida Borges de Araújo, G. Gaffrée Braz, L. Moreira de Lucena, C. Schipper Schwartz, R. Ramoa Pires, A.M. Menezes Aguiar Miranda

## Introduction

Actinomycosis is an infectious disease caused by a gram-positive anaerobic pathogen. Among the clinical manifestations, the cervicofacial region is more affected. Although the periapical location is rare, it is a chronic infection of slow growth. Such infections in the oral cavity begin after the loss of mucosal integrity caused by carious lesions, exposed dental pulp, tooth fracture, and/or dental trauma.

## Case Report

A 29 years old female patient presented with a history of a well-defined radiolucent lesion delimited by a radiopaque, unilocular, oval-shaped halo associated with the radicular apex of 11, 21, and 22. The lesion was found during routine imaging. Therefore, endodontic treatment was performed on the teeth mentioned above, but no periapical repair was observed after 12 months of follow-up. Subsequently, the proposed treatment was apicectomy of elements 21 and 22, excisional biopsy of the periapical lesion, and bone grafting at the surgical site. The histopathology report resulted in periapical Actinomycosis, and the patient was treated with penicillin. The proposed treatment proved to be effective.

## Conclusions

Diagnosing periapical Actinomycosis is challenging, as it produces radiographic findings similar to periapical granuloma or periapical inflammatory cyst. The histopathological evaluation is essential for the correct diagnosis.

# Abstract: Submerged nodular lesion in the buccinator region: a case report

**Authors:** E. Almeida Borges de Araújo, M. Rodrigues Camargos, M. Da Silva Monteiro, G. Laterça da Silva Abre, R. Ramoa Pires, A.M. Menezes Aguiar Miranda

## Introduction

Hemangiomas are benign vascular tumors, most commonly affecting infants’ head and neck region. However, adults can also be affected. Diagnosis is based on clinical history, physical examination, and, when unclear, assisted with ultrasound or MRI.

## Case Report

In March 2021, a 67 years old male with a medical history of systemic arterial hypertension, using losartan and amlodipine daily, presented a lesion on the right cheek mucosa. A flaccid swelling with a slightly yellowish color on the right side of the buccal mucosa was observed on clinical examination. An excisional biopsy was performed, and the histological diagnosis was a lipoma. In January 2022, the patient returned with signs of recurrence of the lesion with pain and paresthesia in the right middle third of the face, with increased extraoral volume. An incisional biopsy was performed to investigate the lesion; however, the histopathological report was compatible with an inflammatory process. In May 2022, the patient maintained a local worsening, with 14 months of evolution approximately. The CT images revealed a heterogeneous lesion, indicating vascular involvement of the facial artery. The proposed plan was an excisional biopsy under general anesthesia. The patient had a more significant volume loss in the transoperative period than expected. Histopathological report of hemangioma resulted after the microscopic analysis of the specimen. After six months of follow-up, the patient presented without recurrence or pain complaints.

## Conclusions

The ideal treatment for a symptomatic hemangioma is often multimodal and may vary depending on each case.

# Abstract: Clinical and microscopic features of mucocutaneous ulcers associated with methotrexate use

**Authors:** L. Ambrósio Lima, M.S. Pereira Sant’ana, C.V. Bardález López De Cáceres, F. Ramôa Pires, F. Paiva Fonseca

## Introduction

The mucocutaneous ulcer (MCU) is a lymphoproliferative disorder (LPS) recognized by WHO in 2017, which is related to Epstein-Barr Virus (EBV) infection in immunosuppressed patients. Females over 70 years old are the most affected patients, and oropharyngeal sites are the most frequently reported. High-grade lymphomas are essential differential diagnoses due to clinical and histological similarities.

## Case Report

In this study, cases of ulcers affecting patients treated with methotrexate (MTX) were retrieved from pathology files of two Brazilian institutions. Clinical data were obtained from patients’ pathology reports. Four cases were obtained, all affecting females with a mean age of 64 and being treated with MTX for rheumatoid arthritis. One of them presented multiple oral lesions. The lower and upper lips, hard palate, and upper right and left alveolar mucosa were the sites affected. Clinically, the lesions presented as a superficial, well-limited ulcer with an erythematous halo, sometimes with an overlying fibrinous layer. Microscopically, the superficial region was ulcerated with an inflammatory infiltrate containing lymphocytes and plasma cells. EBER reaction was positive in one case, leading to an EBV+ mucocutaneous ulcer diagnosis.

## Conclusions

Clinicians and pathologists must be aware of these MTX-associated ulcers, primarily due to their strong similarities with lymphomas.

# Abstract: Functional approach to lingual burning: case presentation

**Authors:** S. Aranda Romo, J.G. Sánchez Medina, F.J. Tejeda Nava, C. Carranza López, A. Campos López

## Introduction

 Early signs of vitamin B12 deficiency without anemia may present with oral manifestations and other vitamins and minerals impacting the oral microbiome. Multidisciplinary management is important in treating patients with vitamin deficiencies.

## Case Report

A 47-year-old female patient with a burning tongue after one year of evolution, a history of hypothyroidism controlled with euthyrox 125mcg and endometriosis, performs a daily physical activity, a diet rich in animal products, insomnia, stress, tiredness, and lack of concentration. Surgical history of cholecystectomy. Intraoral examination revealed erythema on the dorsum of the tongue, atrophy, and hair loss. Laboratory analysis with altered values: Vitamin B12 83pg/mL, Homocysteine 50umol/L, Vitamin D3 22.21ng/ml, Calcium 5mg/ dL, and Candida culture (+843UFC/ml). A diagnosis of Candida albicans dysbiosis, calcium, vitamin D3, and vitamin B12 deficiency without anemia was established. The treatment was carried out with Biogaia-Pro-D® probiotics, vitamin B12, functional nutrition, exercise, stress control, and sleep hygiene. Eubiosis and tongue population were achieved in a week, as well as systemic symptoms. At one year of follow-up, the patient is well.

## Conclusions

The treatment of vitamin deficiencies and oral dysbiosis must be integrative and focused on identifying the origin and resolve it through integrative management to avoid the recurrence of deficiencies and as a preventive strategy for other chronic degenerative diseases.

# Abstract: Pemphigus vulgar in jugal mucosa: a case report

**Authors:** R.G. Barboza, S.M. Amaral, F.R. Pires, T.M.C. Coutinho, M.I. Senne

## Introduction

Pemphigus Vulgaris is a severe and chronic autoimmune disease, clinically manifested by randomly distributed blistering lesions on the skin and mucosa, resulting from IgG autoantibodies against glycoproteins 1 and 3, which cause acantholysis of the epithelium. The oral regions frequently affected are the palate, labial and jugal mucosa, tongue, and gums. In addition, these lesions can precede, to a great extent, the appearance of cutaneous manifestations, which confers to the dental surgeon the responsibility of early diagnosis of this disease.

## Case Report

We reported a clinical case of Pemphigus Vulgaris in a 61-year-old patient, referred to the UNESA stomatology clinic, who presented a single ulcerated region in the jugal mucosa covered by a white pseudomembrane, with an extension of 3.5 cm in its greatest diameter, up to the bottom of the vestibule and alveolar ridge and positive Nikolsky’s sign. A perilesional incisional biopsy was performed with the hypothesis of pemphigus Vulgaris, benign pemphigoid, and erosive lichen planus. The surgical specimen was sent to the Oral Anatomic Pathology Service of the UERJ, revealing an intraepithelial cleft with acantholysis of keratinocytes, loss of adhesion of desmosomes, and retraction of tonofilaments giving rise to acantholytic cells called Tzanck cells, with a final diagnosis of Pemphigus Vulgaris. The patient was referred to the dermatology department of SUS.

## Conclusions

A representative sample obtained by incisional biopsy and adequate clinicopathological correlation is essential in diagnosing Pemphigus Vulgaris.

# Abstract: Pyogenic granuloma of the mandible: a case report

**Authors:** A. Benitez, C. Servín, N. Rivas Torres

## Introduction

Pyogenic granulomas are reactive hyperplastic lesions that appear red and with a softer consistency. Microscopic features are multiple capillary-sized vessels embedded within a loose fibrous connective tissue and a dense inflammatory infiltrate. It is relatively common, and although its origin remains uncertain, it is associated with local irritants.

## Case Report

A 45 years old female patient was evaluated at the School of Dentistry of the Universidad del Norte. During a physical examination, a pale pink nodule with reddish regions measuring approximately 2,5 cm in diameter was observed in the right premolar region of the mandible. The patient noticed the lesion about six months ago and related painful symptoms during palpation. An excisional biopsy was performed, and the specimen was sent for histopathological analysis. The slides showed granulation tissue with abundant blood vessels, fibroblast proliferation, inflammatory infiltration with lymphocytes, plasma cells, and neutrophils, surrounded by hyperplastic squamous epithelium. Therefore, the findings were compatible with Pyogenic Granuloma. Post-operatory control after seven days showed satisfactory healing of the surgical area.

## Conclusions

Even considering the pyogenic granuloma is a benign lesion, it may reach considerable size, interfering with the patient’s well-being. Furthermore, it is essential to dismiss more aggressive lesions, and histopathological analysis is mandatory.

# Abstract: Primary intraosseous B-cell lymphoblastic lymphoma of the mandible: a case report

**Authors:** C.P. Caamaño Martínez, V.E. Palacios Inostroza, E. Báez Rivas

## Introduction

Lymphoblastic lymphoma (LBL) is a malignant neoplasm that originates from precursors of B or T lymphocytes and constitutes approximately 2% of all non- Hodgkin lymphomas. They are infrequent in the oral cavity, even more so if it is an extra-nodal primary presentation. In this location, neither the clinical features nor the radiological appearance is pathognomonic and can represent significant diagnostic problems.

## Case Report

We present the case of a seven-year-old female patient with a slowly enlarging painless right mandibular mass of two weeks of evolution. Radiographic studies showed an infiltrative hypodense area involving the right mandibular body, compromised teeth in formation, and vestibular and lingual cortical. Histological examination revealed a malignant neoplasm with a proliferation of round, small, blue morphology cells. In addition, immunohistochemical markers for TdT and PAX5 were positive. The diagnosis was B-cell lymphoblastic lymphoma and complementary studies confirmed only mandibular involvement without infiltration to other organs.

## Conclusions

The presentation of this case seeks to describe a rare neoplasm in the oral cavity, including clinical and radiographic presentation, histological characteristics, and immunological profile. The importance of an early and accurate diagnosis of this entity is emphasized due to the impact on patient survival.

# Abstract: From lichen and proliferative verrucous leukoplakia towards malignant transformation. Six years of onco-stomatological follow-up

**Authors:** A. Castrillo, V. Flück, C. Giacco, J. Giménez

## Introduction

A broad spectrum of multifocal white lesions of the oral mucosa is generally associated with potentially malignant disorders such as oral lichen planus, oral lichenoid lesions, and proliferative verrucous leukoplakia. They are dynamic entities that can acquire different clinical and histopathological characteristics during their evolution and carry a greater or lesser extent risk of malignant transformation.

## Case Report

A 71-year-old male patient was referred to HMOMC with white, warty, multifocal vegetative lesions and histopathological diagnoses of oral lichen, proliferative verrucous leukoplakia, verrucous carcinoma, and carcinoma in situ. Among the different locations, the upper labial mucosa, upper and lower buccal gingiva, left buccal mucosa, and upper and lower residual ridges stand out, observing areas of stains and keratosis with areas of healthy mucosa and vegetative/warty areas that emanate keratin under pressure. Different treatment approaches were carried out according to the clinicalpathological diagnosis, among which we highlight chemotherapy with methotrexate and conservative surgeries. Having as a common denominator strict follow-up for six years to date with disease control.

## Conclusions

Different clinical situations can be challenging, especially when it becomes difficult to distinguish oral lichen and proliferative verrucous leukoplakia. Because these persistent multifocal lesions are refractory to interventions, a correct interpretation of changes in these lesions’ clinical and pathological features is mandatory. Therefore, close follow-up and biopsies are the most appropriate approach to detecting early malignant transformation.

# Abstract: Maxillary osteomyelitis secondary to mucormycosis, a sequel of COVID-19: a clinical case report

**Authors:** F.M. Contreras Jimenez, H. Barria Ângulo, J.I. Ore De la Cruz, H. Ochoa Huamán

## Introduction

One of the consequences of COVID-19 is the incidence of mucormycosis in the jaws and subsequent osteomyelitis in patients with undiagnosed or uncontrolled comorbidities, such as diabetes mellitus and associated immunosuppression.

## Case Report

A 52-year-old male patient with a history of COVID-19 two months ago presented a painful ulcerative lesion of insidious onset in the palatal raphe measuring approximately 2 mm. He referred to numbness of the palatal region of one month of evolution. During the physical examination, purulent content, multiple pustules in the anterior maxillary buccal mucosa, and mobility of upper anterior teeth were observed. The CT revealed isodense bilateral images in maxillary and ethmoidal sinuses, bone sequestrations, and partial loss of anterior vestibular cortical bone. Laboratory tests revealed no abnormality, except for HbH1c: 10.2gr/dl. The patient was hospitalized for control of newly diagnosed diabetes mellitus. Maxillary incisional biopsy was performed, and microscopic analysis showed a mixed inflammatory infiltrate, fibrin deposits with eosinophilic and birefringent ribbon-like hyphae, branched at right angles, compatible with maxillary osteomyelitis secondary to mucormycosis. The treatment started with antifungal and intravenous antibiotics, followed by surgical cleaning under general anesthesia. The patient progressed favorably.

## Conclusions

Immunosuppression resulting from COVID-19 and/or uncontrolled systemic diseases can condition the appearance of rare opportunistic microorganisms causing infections such as mucormycosis. Early diagnosis and treatment make a difference in the morbidity and mortality of patients.

# Abstract: Glandular odontogenic cyst, diagnostic importance for surgical conduct: a case report

**Authors:** F.M. Contreras Jimenez, H. Barria Ângulo, J.I. Ore De la Cruz, H. Ochoa Huamán, J. Nuñez Villalba

## Introduction

The glandular odontogenic cyst is a rare pathology of the jaws, with clinical, imaging, and recurrent characteristics similar to ameloblastoma and keratocyst. Microscopically presents similar findings to mucoepidermoid carcinoma. Therefore, the present case emphasizes the differential diagnostic importance for the best treatment of the patient.

## Case Report

A 27-year-old female patient presented with a cystic lesion found by radiographic image while undergoing endodontic treatment. The intraoral examination revealed a slight volume increase in the left retromolar region adjacent to the second molar. The radiographic images showed a well-defined multilocular radiolucent lesion in the ramus and left mandibular angle of approximately 5x3 cm with the absence of tooth 3.8. The imaging features were compatible with keratocyst or ameloblastoma. A biopsy was performed, and during the anatomopathological analysis was observed a flat polystratified epithelium with cuboidal cells presenting intraepithelial microcysts, and some ciliated projections without cellular atypia. The diagnosis was compatible with glandular odontogenic cyst. The patient was treated by conservative cystic enucleation and peripheral osteotomy. After four years of follow-up, the patient presents favorable evolution without signs of recurrence.

## Conclusions

The anatomopathological diagnosis of maxillary cysts and tumors is essential in pathologies with similar clinical and radiographic features because it defines the surgical treatment for the patient.

# Abstract: Odontogenic myxoma with radiographic aspects in sunlight

**Authors:** R.B. De Araújo Cunha, G.R.W. De Oliveira, A.C.L. Da Silva Leonel, D.E. Da Cruz Perez, E.J. De Amorim Carvalho

## Introduction

Odontogenic Myxomas are found mainly in young adults with a mean age between 25 and 30 years, but they can occur in a wide age range without gender predilection. The mandible is more commonly affected than the maxilla, and minor lesions may be asymptomatic. This work aims to report a clinical case of Odontogenic Myxoma with radiographic aspects in sunlight.

## Case Report

A 21-year-old female patient sought a diagnostic service reporting an increase in volume in the left hemiface for five months. On extraoral clinical examination, it was possible to notice an increased volume in the left hemimandible of firm consistency on palpation. On intraoral examination, he had a vestibular bulge with intact overlying mucosa. The imaging exams were ordered for diagnostic purposes. Cone-beam computed tomography revealed a well-delimited, multilocular hypodense image extending from the premolars to the mandibular notch. It was possible to notice the presence of periosteal reaction in the buccal bone plate with aspects of sun rays. Because of the radiographic characteristics, the diagnostic hypotheses were osteosarcoma and Odontogenic Myxoma.

## Conclusions

The patient underwent hemimandibulectomy, and the surgical specimen was sent for analysis. In the histological examination was observed fusiform and stellate cells arranged in a loose myxomatous connective tissue. The final diagnosis was Odontogenic Myxoma. After one year of treatment, no signs of lesion recurrence were observed.

# Abstract: Oral lymphoepithelial cyst

**Authors:** E. De la Rosa García, J. Maldonado Mendoza

## Introduction

The oral lymphoepithelial cyst (OLC) is a developmental cyst with a frequency of <1% of all intraoral cysts. The OLC locates in the posterior part of the oral cavity, is asymptomatic, and has unclear pathogenesis. Therefore, other entities with lymphoid tissue should be considered differential diagnoses.

## Case Report

A 25-year-old woman presented at the Oral Pathology and Medicine Clinic of the Universidad Autónoma Metropolitana, referred by a Dentist for a sore in her tongue. During the intraoral examination, we observed a round yellowish-white papule with a smooth surface, sessile, firm on palpation, 3 x 2 x 3 mm in size, and years of evolution. This papule was located in the posterior third of the tongue. After a routine serological test evaluation, an excisional biopsy of the lesion was scheduled. Microscopic findings showed a cystic cavity lined by stratified squamous epithelium without rete ridges. This epithelium was parakeratinized, with desquamated epithelial cells filling the cyst lumen. The underlying capsule showed mature connective tissue with lymphoid tissue with a follicular pattern.

## Conclusions

The present case represents the demographic and clinical characteristics previously reported for OLC; however, developmental entities with lymphoid tissue, such as Lymphoid Hyperplasia and Dermoid cyst, should be considered within the differential diagnosis.

# Abstract: Odontogenic keratocyst in the anterior region of the jaw: a case report

**Authors:** G.R.W. De Oliveira, R.B. De Araújo Cunha, A.C.L. Da Silva Leonel, D.E. Da Cruz Perez, E.J. De Amorim Carvalho

## Introduction

This work aims to report a clinical case of Odontogenic Keratocyst located in the anterior region of the mandible.

## Case Report

A 33-year-old male patient attends the diagnostic service due to dental mobility of teeth 31 and 41, associated with pressure on the same teeth. On intraoral examination, she had a vestibular bulge with intact overlying mucosa. The pulp vitality test of the affected teeth was positive. Imaging tests were requested for diagnostic purposes. Cone beam computed tomography revealed a well-demarcated unilocular hypodense image located between teeth 31 and 41, causing root divergence. It was possible to note the presence of thinning and slight bulging of the buccal and lingual cortices, in addition to rupture of the buccal cortical. Based on the radiographic characteristics, the diagnostic hypotheses were lateral periodontal cyst, odontogenic keratocyst, and adenomatoid odontogenic tumor extrafollicular variant. The patient underwent an incisional biopsy, and the surgical fragments were sent for analysis. Histological examination revealed a cystic cavity lined by corrugated and parakeratinized stratified squamous epithelium, with basal cuboidal cells layer arranged in a palisade. Also, fine capsule of fibrous connective tissue.

## Conclusions

The final diagnosis was Odontogenic Keratocyst. After diagnosis, the patient underwent complete surgical excision and is under follow-up with no clinical and radiographic recurrence history.

# Abstract: Surgical management of mandibular ameloblastoma with rapid evolution: a case report

**Authors:** K. Ferreira Farias Catarino, G. Alves Ribeiro, D. Zimmermann, E. Pantoja, F. Ramoa Pires, A.M. Menezes Aguiar Miranda

## Introduction

Ameloblastomas are benign odontogenic neoplasms of epithelial origin presenting local aggressive behavior. They affect the mandible predominantly and can reach varying proportions depending on the Evolution time. These tumors are locally invasive, expansive, and may have jagged margins and a high recurrence rate. Consequently, it results in severe clinical implications. In this sense, the purpose was to describe surgical management in patients with rapidly evolving ameloblastoma.

## Case Report

This report describes a case of a 19-year-old man who presented an extensive radiolucent lesion in the left mandible with a significant increase in volume for two months without reporting any pain. The patient denies comorbidities, allergies, and medication use. Due to the aggressive nature of this lesion, removal of the affected soft tissue in the intraoral region was performed, followed by extraoral surgical access via high cervicotomy to perform segmental resection with margin as a treatment option associated with reconstruction with an internal fixation system plate. rigid 2.4 in order to maintain the mandibular contour.

## Conclusions

In this context, although ameloblastoma is benign, its aggressive nature can limit the patient’s quality of life. So, the local invasion features of this tumor make the radical treatment more indicated in most cases by reducing the possibility of future recurrence.

# Abstract: Adjuvant therapies in the treatment of neurosensory disorders after enucleation of odontogenic keratocyst: a case report

**Authors:** K. Ferreira Farias Catarino, A.M. Menezes Aguiar Miranda, F. Ramoa Pires, A. Miranda Rocha, F. Chiella Büchele, M. Rodrigues Camargos

## Introduction

Odontogenic keratocyst is a benign but aggressive lesion with a high recurrence rate. It affects the gnathic bones with prevalence in the posterior body and mandibular ramus. One of the treatment options is enucleation associated with peripheral osteotomy. However, depending on the proportions of the lesion, the use of extraoral access is necessary. In this context, a possible complication is a damage to the motor and sensory nerves of the oral and maxillofacial complex.

## Case Report

The clinical case refers to a 24 years old female patient with a history of systemic arterial hypertension and obesity. The patient was diagnosed with extensive odontogenic keratocyst in the right mandibular angle and ramus region. Enucleation of the lesion associated with peripheral osteotomy was performed. In the postoperative period, the patient evolved with paresis of the marginal nerve of the mandible and paresthesia of the inferior alveolar nerve. In this case, Low-Level Laser Therapy (LLLT) is a therapy for sensory disorders that accelerates the regeneration of damaged nerve tissue, stimulating adjacent tissues and biomodulating the nerve response. It’s been shown that, when associated with pyrimidine ribonucleotides, a neurotrophic medication, such as ETNA®, helps in the rehabilitation process by acting on the metabolism of the nervous system.

## Conclusions

It is important to highlight that the multimodal approach to treating sensorineural disorders through LLLT jointly with ETNA® seems to be effective in resolving sensorineural disorders caused by surgical trauma.

# Abstract: Multifocal proliferative leukoplakia and oral lichen planus: Is it the evolution from one entity to another or a different clinical phenotype?

**Authors:** G. Gilligan, F. Frascaroli, J.C. Romero, E. Piemonte, R. Panico

## Introduction

Multifocal Proliferative Leukoplakia (MPL) is considered the Oral Potentially Malignant Disorder (OPMD) with the highest rate of malignant transformation. Some studies have indicated that it may initially present with a reticular appearance, mimicking oral lichen planus (OLP), or it may be an evolutionary phenomenon that occurs in some patients with OLP. Objectives: To report a clinical case where both entities’ clinicalpathological characteristics and criteria coexist, considered OPMD by the WHO.

## Case Report

A 48-years old male who presented in the framework of the Campaign for Prevention and Early Diagnosis of Oral Cancer in Córdoba, Argentina, with multiple white lesions of three years of evolution. Regarding habits, the patient was a heavy consumer of tobacco and a nonalcohol drinker. The patient was diagnosed with OLP by another Service five years earlier. On consultation, he presented bilateral lesions on the lateral tongue border compatible with OLP. The biopsy of these reticular areas showed the presence of hyperkeratosis and an interface infiltrate compatible with OLP. In addition, the patient presented white plaques with warty areas on both buccal mucosa and gingiva compatible with PML. Serial biopsies of these sectors showed hyperkeratosis with dysplastic features. For the final diagnosis, the current criteria for PML were met.

## Conclusions

Although it is difficult to elucidate whether PML initially manifests as OLP or if a subset of OLP evolves into PML, the most relevant aspect of oral cancer prevention is close follow-up and monitoring of patients with reticular white plaques.

# Abstract: Multiple myeloma atypical presentation in sunrise image. Case report

**Authors:** J. Giménez, A. Castrillo, V. Flück, M. De Dios Soler, C. Giacco

## Introduction

Multiple myeloma (MM) is a plasma cell neoplasm characterized by the proliferation of a single clone of plasma cells that produce circulating immunoglobulins. The most commonly observed radiographic image is punch-shaped and can manifest as a single or multiple lesions. Here we present a case of mandibular MM with an atypical radiographic image.

## Case Report

We present a 46-year-old male patient who attended the HMOMC for a lesion of more than one year of evolution. On extraoral clinical inspection, we observed left facial asymmetry, intraorally in the left hemimandibula ulcerated pink tumor larger than 2 cm involving the vestibule. The orthopantomography showed a radiolucent image with poorly defined borders in the left mandibular body and a “rising sun” image towards the basal area. A biopsy and pathological anatomy were performed, resulting in a plasma cell neoplasm. The patient was referred to the oncohaematology department, where the entire skeleton was studied, and bone marrow biopsy, PET scan, and laboratory tests confirmed the presence of MM.

## Conclusions

MM can present radiographically in 3 different ways, the most common presentation being observed as punch-shaped images with well-defined borders. In this case, the “reactive” image on the basal mandibular area is noteworthy, making it necessary to consider differential diagnoses with other malignant neoplasms. It is important to remember the possible radiographic presentations of MM and to stress the importance of anatomopathological evaluation in patients with atypical symptoms and presentations.

# Abstract: Therapeutic alternatives for drug-associated maxillary osteonecrosis (MRONJ): reports of two clinical cases

**Authors:** N. Leonardi, G. Gilligan, E. Piemonte, R. Panico

## Introduction

Drug-associated Maxillary Osteonecrosis is one of the most relevant adverse effects in treatment with antiresorptive drugs such as bisphosphonates and Denosumab. Pharmacological, conservative, and surgical treatments are described. Objectives: The aim of this study is to report two clinical cases of MRONJ treated with two different therapeutic protocols.

## Case Report

Case 1: a 68-year-old female patient was referred for pain associated with an active and suppurating mucocutaneous fistula with a history of oncology (breast carcinoma). The patient had been treated with Zoledronic acid and Alendronate for 2 and 5 years, respectively. Clinically, an ulcerated lesion was observed with necrotic bone tissue exposed on the left mandibular ridge, posterior sector. A local treatment protocol was applied with conservative management. Case 2: a 63-year-old female with an underlying pathology of osteoporosis was treated with Ibandronate + Denosumab for 18 years. Clinically, an ulcerated lesion was observed with exposed necrotic bone tissue on the left mandibular ridge, posterior sector. A surgical treatment protocol was applied to obtain and use plasma rich in fibrin (PRF).

## Conclusions

Patients who develop oral clinical pictures of great morbidities, such as MRONJ, may see their quality of life conditioned and suffer a worsening of their underlying pathology. Therefore, the treatment of MRONJ depends on the stage of the disease, and its success depends on interdisciplinary management and strict medical and dental clinical follow-up.

# Abstract: Clinical manifestations and new evidences of genetic heterogeneity for hereditary gingival fibromatosis

**Authors:** R.A. Machado, R.S. Andrade, S.P.B. Pêgo, A.C.V. Krepischi, R.D. Coletta, H. Martelli-Júnior

## Introduction

Hereditary gingival fibromatosis (HGF) is an uncommon genetic condition characterized by slow and progressive fibrous, non-hemorrhagic and painless growth of the gingival tissues due to the increased deposition of collagen and other macromolecules in the extracellular matrix. HGF occurs in approximately 1:750,000 live births and can be transmitted as an autosomal dominant trait or recessive inheritance. To date, five loci (2p22.1, 2p23.3–p22.3, 4q12, 5q13–q22, and 11p15) and three genes [REST (RE1-silencing transcription factor), SOS1 (Son-of-Sevenless-1) and ZNF862 (zinc finger protein 862 genes)] has been associated with the HGF.

## Case Report

In the current study, we report 13 individuals with HGF from 4 unrelated families. The clinical presentation of gingival overgrowth was similar among all affected individuals; however, differences were observed in the intensity of the overgrowth, even in members of the same family. Five patients showed severe symptoms, two patients showed moderate, and six showed mild symptoms. Whole-exome sequencing (WES) revealed four different mutations, including two frameshifts and two missense variants in CD36, REST, SOS1, and ALK genes. In the first family was found the c.1133G>T mutation in CD36: the second family, a deletion in REST (c.1491_1492delAG). In the third, an insertion in the c.3265 position in SOS1. And in the last family, the missense mutation (c.361C>T) in ALK.

## Conclusions

Together, these results suggest a pattern of heterogeneity in the clinical manifestation, inheritance, and mutation patterns in each family affected by HGF.

# Abstract: Low-grade mucoepidermoid carcinoma in a young adult: a case report

**Authors:** A. Moriya Agüero, B. Amarilla Cáceres, J. Gamarra Insfrán, M.C. González Galván

## Introduction

Mucoepidermoid carcinoma represents one of the most frequent malignant neoplasms in the salivary glands. It occurs in major salivary glands such as the parotid gland, and in the case of minor salivary glands, the most commonly affected site is the palate. It is found between the fourth and sixth decade of life, with a predominance in women.

## Case Report

A 22-year-old male patient with unremarkable medical history attended the School of Dentistry of the National University of Asunción referred by his dentist after noticing a slight discoloration different from the adjacent mucosa. During the anamnesis, the patient related occasional discomfort in the hard palate area for one year. In the intraoral examination, an ovoid lesion was observed on the left side of the hard palate, presenting purplish color, measuring 1 cm in diameter and depressible to palpation. A panoramic x-ray and a computerized axial tomography showed no bone alterations. An incisional biopsy was performed, and the histopathological study resulted in a low-grade mucoepidermoid carcinoma.

## Conclusions

This specific carcinoma is atypical to be found in young patients, compared to the age range in which it usually appears. In any case, it should be considered in the differential diagnosis due to its clinical characteristics.

# Abstract: Intraoral monophasic synovial sarcoma: a case report

**Authors:** E. Ojeda de Mestral, F. Perrota, F. Bareiro, C.R. Invernizzi Mendoza

## Introduction

Synovial sarcoma is classified as a malignant soft tissue tumor that represents between 5.6% and 10% of all soft tissue sarcomas. The head and neck are affected in 3-10% of cases, which is rare in the oral cavity.

## Case Report

A 23-year-old woman with significant facial asymmetry attended the consultation. During intraoral examination a vestibular swelling of the left lower hemiarch and absence of teeth were observed. Computed tomography revealed a heterogeneous lesion measuring approximately 50x38x65 mm on the left side of the mandible, causing extensive bone destruction and invading adjacent soft tissues. An incisional biopsy was performed and histopathological results reported a high-grade conventional osteosarcoma. Subsequently, hemi-mandibulectomy was performed with a reconstruction plate plus condyle under general anesthesia. The specimen was sent for microscopic analysis, and the diagnosis was Monophasic Synovial Sarcoma, confirmed by immunohistochemistry. The evolution of the patient was favorable and she is currently under oncology follow-up.

## Conclusions

The intraoral location of Synovial Sarcoma is infrequent, and only 5 cases of monophasic type have been described in the literature. Despite its rarity in the oral cavity, dental surgeons must pay attention to this entity. Accurate diagnosis and multidisciplinary management of the patient are essential in these cases.

# Abstract: Oral leiomyoma: rare entity and the importance of a correct diagnosis

**Authors:** M. Orellana Higueros, O. Toralla de León, C. López Acevedo

## Introduction

Leiomyomas are benign smooth muscle tumors that commonly affect the uterus, skin, and gastrointestinal tract. They represent only 0.42% of all soft tissue lesions reported in the oral cavity and might originate from the salivary glands’ vascular walls or excretory ducts.

## Case Report

A 46-year-old woman attends the Oral Medicine Clinic and Histopathological Laboratory “Dr. César López Acevedo” in FOUSAC-Guatemala. The intraoral clinical examination revealed a painful, ovoid-shaped nodule located in the lower labial mucosa measuring 7 x 7mm, presenting a smooth surface, soft consistency, the same color as the adjacent mucosa, and with unknown evolution time. The differential diagnoses were mesenchymal lesions vs. salivary gland neoplasms. An excisional biopsy was performed, and microscopic evaluation evidenced a well-delimited solid neoplasm of spindle cells arranged in intercalated and elongated fascicles presenting oval nuclei in blunt ends. Immunohistochemistry results showed positivity for AML and Demin; S-100 was negative. Therefore, the diagnosis was solid leiomyoma.

## Conclusions

Despite its rarity, leiomyoma should be considered a differential diagnosis for benign lip lesions. Knowing this entity’s clinical, histopathological and immunohistochemical features can help with accurate diagnosis and treatment.

# Abstract: Oral manifestations of Chikungunya

**Authors:** S.P.B. Pêgo, L.C. Farias, W.C. Almeida, D.R.B. Martelli, A.M.B. Paula, H. Martelli-Júnior

## Introduction

Chikungunya fever is caused by the Chikungunya vírus, transmitted by mosquitoes, Aedes aegypth, and Aedes albopictus. The most common symptoms are fever, asthenia, arthralgia, and myalgia.

## Case Report

A 22-year-old Brazilian male was referred to the Oral Medicine service due to the appearance of asymptomatic red patches in the oral mucosa. The patient reported remission and recurrence of red spots on the palate approximately 60 days before the initial consultation. He also reported feelings of dry mouth, bitter taste, fever, and joint pain, especially feet and hands. The patient did not report alterations on the skin or other mucous membranes. Other relatives did not present this clinical condition. The medical history was unremarkable, and the patient did not remember local trauma. On the physical evaluation, there was no cervical lymphadenopathy or skin patches. Intraoral examination revealed multiple red patches on the hard palate, lip mucosa, and cheek mucosa measuring approximately 5mm. The medical support requested an RT-PCR test to detect the Chikungunya virus, which resulted in a positive result. We perform an incisional biopsy on the mucosa of the upper lip. The microscopic findings were compatible with a nonspecific chronic inflammatory process, compatible with a case of viral infection. Based on the patient’s history, physical examination, and laboratory findings, the diagnosis was Chikungunya fever. The patient has been followed up.

## Conclusions

The clinical behavior related to the remission and recurrence of oral lesions need to be better understood.

# Abstract: Oral manifestation of extranodal NK/T cell lymphoma

**Authors:** M.S. Pereira Sant’ana, C.V. Bardález López de Cáceres, L. Ambrosio Lima, J.C. Tanos De Lacerda, R. Gonçalves Resende, F. Paiva Fonseca

## Introduction

Extranodal NK/T-cell lymphoma (ENKTCL) is an aggressive lymphoid neoplasm with geographic predilection, including in Latin American countries. Clinically, ENKTCL is strongly associated with Epstein Barr Virus (EBV) infection and appears as a necrotic ulcer affecting upper airway structures.

## Case Report

We present a case of a 90 years old male exhibiting a painful and bleeding ulcer in the hard palate, extending to the soft palate and alveolar ridge, with a necrotic center and erythematous borders. Extra oral examination revealed unilateral cervical lymphadenopathy. An incisional biopsy was performed, and the specimen observed under light microscopy showed a diffuse proliferation of small lymphoid cells, vascular invasion and destruction, and necrosis. Immunohistochemistry was positive for CD3, Granzyme B, Perforin, and a high Ki67 index (85-90%). In situ hybridization for the detection of EBV was diffusely positive. Therefore, a final diagnosis of extranodal NK/T cell lymphoma was established, and the patient was referred for oncology service care.

## Conclusions

Despite its distinctive clinical presentation and microscopic features, diagnosis of extranodal NK/T cell lymphoma may be very challenging.

# Abstract: Orofacial leishmaniasis: the importance of early diagnosis in dental care

**Authors:** D.B. Pereira, K.P.M. Lelis, L.C. Machado, F.S.N. Diogo, K.T. Bezerra, J.N.P. Barros

## Introduction

Leishmaniasis is an infectious disease caused by a protozoan and transmitted by the bite of an infected insect. Its clinical manifestations are lesions in the mucosa or skin of the head and neck region. The cutaneous form begins with the development of a macula, which will evolve into a papular lesion and progressively increase and form an ulcer. Destructive lesions in the mucosa of the upper airways characterize Mucosal Leishmaniasis. Both forms are a challenge for the scientific community as they present a series of findings and can mimic other infectious and malignant diseases.

## Case Report

We report a clinical case of a 56-year-old melanoderma patient who sought the dental emergency service of a public hospital in Rio de Janeiro, complaining of “skin and mouth lesions” of approximately two months of evolution. While taking his medical history, he mentioned smoking and alcoholism, denying allergies and other pathologies. However, at the time of the examination, he still reported pain, cough, and difficulty swallowing and breathing. After laboratory and imaging tests, we performed an incisional biopsy, which showed the immunological findings of conventional plasma cell infiltrates and granuloma formation.

## Conclusions

This disease should be part of the differential diagnosis of lesions that cover the field of work of the dental surgeon, allowing for early diagnosis and prompt referral to appropriate medical treatment.

# Abstract: Mucoepidermoid carcinoma in the jugal mucosa: A case report

**Authors:** L.L. Rentes, S.M. Amaral, M.I. Senne, F.R. Pires

## Introduction

Mucoepidermoid carcinoma is a malignant tumor of the salivary glands that can affect patients in a wide age range and shows a predilection for the parotid glands, followed by the minor salivary glands of the palate. It is a progressive and asymptomatic swelling but can present a more aggressive behavior. Treatment consists of surgical resection and postoperative radiotherapy in more aggressive tumors.

## Case Report

We aim to report a case of mucoepidermoid carcinoma with non-aggressive clinical features and slow evolution, delaying its diagnosis. A 46-year-old female was referred to the UNESA Dental Clinic with a complaint of swelling in the posterior region of the mouth, perceived ten years ago. However, it began to cause discomfort in the last two years due to the orthodontic treatment. The medical history revealed nothing of note. In the intraoral physical examination, the right cheek mucosa was intact. However, during palpation, a firm, submerged nodule was observed close to the bottom of the vestibule of tooth 45 (absent), fixed, measuring 1.5 cm in its largest diameter. An excisional biopsy was performed with an initial diagnosis of lipoma or irritating fibroma. The surgical specimen was sent to the Laboratory of Oral Pathology at FO/UERJ, and the anatomopathological examination revealed the diagnosis of Mucoepidermoid Carcinoma. The patient underwent surgical treatment, and the final specimen confirmed the diagnosis of Mucoepidermoid Carcinoma.

## Conclusions

Conducting the clinical examination was essential for both diagnosis and prognosis, considering the time of evolution without any previous intervention.

# Abstract: Pleomorphic adenoma with giant recurrent cystic pattern

**Authors:** K. Rodriguez Ibazetta, D. Mamani Cabezas, S. Sacsaquispe Contreras

## Introduction

Pleomorphic adenoma is a benign salivary gland tumor, and the palate is the most common location when it affects minor salivary glands (10%). The size varies from 2 to 6 cm in diameter, and local recurrence is 1-5% of cases. The average interval between excision of the lesion and recurrence is 7-10 years.

## Case Report

42-year-old female patient with a history of pleomorphic adenoma surgery six years ago in the left palatine region presented with facial asymmetry due to increased volume in the left genian region with effacement of the lip-genian groove. On intraoral examination, she presented a tumor on the hard palate with a soft consistency, not ulcerated, and mild pain of size approximately 6x5x2.5cm. Imaging examination showed an osteolytic lesion with peripheral protrusion occupying the maxillary sinus and left nasal fossa, unilocular, with defined limits and partially corticated edges, which caused the displacement of the posterior and inferior wall of the maxillary sinus. An excisional biopsy was performed, and the histopathological examination showed an epithelial proliferation consisting of two cells population, areas of chondromyxoid stroma, and cystic degeneration. In addition, the epithelial component presents increased cellularity, nuclear atypia, and mitotic activity in some areas. It concludes with a cystic pleomorphic adenoma of atypical presentation.

## Conclusions

Pleomorphic adenoma can recur, be aggressive, fastgrowing, and present certain histopathologic features that could mimic malignancy, as the case presented.

# Abstract: Importance of the correct diagnosis of maxillary osteonecrosis

**Authors:** J.A. Ruiz Roca, N. Soto Ayén, F. Molero Galera, F.J. Gómez García, P. López Jornet

## Introduction

Treatment with antiresorptive drugs and antiangiogenic agents is increasingly used, sometimes producing numerous side effects at the cavity level. Osteonecrosis of the jaws is a problematic pathology to solve in daily clinical practice. The objective is to present a clinical case with an initial diagnosis of osteonecrosis of the jaws.

## Case Report

A 63-year-old patient is referred to the Oral Medicine Unit. As medical history, he presents hypertension, treated with antihypertensive drugs, Condrosanâ for joint problems, and no known toxic habits. Three months before, the patient had been rehabilitated with implants in 4.5 and 4.6. In the treatment area, a rough, white-brownish structure was located in the lingual attached gingiva, distal to the restoration, with a presumptive diagnosis of osteonecrosis. Complementary tests (CBCT) were performed, and the structure in question was found to be a root remnant.

## Conclusions

For correct diagnosis, it is essential to take a clinical history and use the appropriate tools, in this case, clinical judgment and imaging tests. Knowing the different diagnostic criteria and pathologic antecedents is necessary to make a good differential diagnosis.

# Abstract: Heck’s disease in the elderly patient

**Authors:** C.G. Ruiz Rojas, F.S. Cerron Soto, S.J. Sacsaquispe Contreras

## Introduction

Heck’s disease, which corresponds to focal epithelial hyperplasia, is a proliferation of the squamous epithelium attributed mainly to HPV 13 and 32. It arises predominantly in children and adolescents.

## Case Report

A 61-year-old man with a pathological history of HSV- 2, gonorrhea, and stroke, went to the clinic for comprehensive dental treatment, where several asymptomatic papules were found on the oral mucosa, with an undetermined time of illness. As history, he reported having been an active smoker for many years (4 cigarettes/ day). Clinically, multiple papules were observed on the lower labial mucosa, bilateral buccal mucosa, and ventral lingual aspect. Presumptive diagnoses were focal epithelial hyperplasia, syphilis mucosal patch, frictional keratosis, and moriscatio bucarum. A complete blood count was indicated, presenting typical values and non-reactive syphilis. An excisional biopsy of some papules of the lower labial mucosa and buccal mucosa was performed. Histopathological examination under HE showed oral mucosa with squamous epithelium with marked acanthosis and pseudoepitheliomatous hyperplasia with the presence of koilocytes, and lamina propria with mild chronic inflammatory infiltrate. The HPV marker was positive. Diagnosis: focal epithelial hyperplasia. The follow-up was carried out after two weeks, showing a favorable evolution. Currently, follow- ups and controls are carried out.

## Conclusions

Focal epithelial hyperplasia is a rare pathology that could occur in adults, so it should be considered a differential diagnosis. Those lesions that do not regress spontaneously can be surgically removed if it represents aesthetic involvement or recurrent trauma.

# Abstract: Oral squamous cell carcinoma, sarcomatoid variant of the tongue. Instituto de Prevision Social. Case report

**Authors:** E.M. Samudio Scavone

## Introduction

Squamous cell carcinoma (SCC) is a relatively frequently observed pathology in Paraguay’s hospitals and dental offices. In most cases, it is not diagnosed early and treated, and it presents a severe risk to people’s health. The sarcomatoid variety represents a rare and more aggressive subtype than its conventional presentation, with high recurrence rates and lymphatic metastases. Exposure to previous radiation represents one of its main triggering factors.

## Case Report

An 83-year-old male presented a painful reddish multilobulated mass with spontaneous bleeding and constant burning sensation. An incisional biopsy was performed, which resulted in a sarcomatoid oral squamous cell carcinoma. The patient underwent a hemiglossectomy, cervical dissection, and six cycles of chemotherapy with cisplatin. After four months, the patient relapsed, did not respond to the treatment and passed away nine months after the surgical procedure.

## Conclusions

It is an illness with a multidisciplinary approach, where the dental surgeon or dentist with an oral medicine degree plays a fundamental role in its early diagnosis and in the identification of the various potentially malignant disorders, in addition to promoting prevention campaigns in oral cancer or in (PMD) that in the long run, it has a positive impact on the Paraguayan population that many times due to lack of interest or ignorance of an examination of the oral cavity could change the course of this disease.

# Abstract: Pigmented melanoma of the tongue, Instituto de Prevision Social. Case report

**Authors:** E.M. Samudio Scavone

## Introduction

Oral mucosal melanoma (OMM) is rare and represents 0.2 - 8% of all melanomas. Its aggressiveness and prognosis have mostly stayed the same in the past few years. To consider it an actual primary lesion of the oral cavity, we must identify any other origin because it will define the therapeutic and prognostic approach; differentiating it from other pigmented lesions is a real challenge. Its etiopathogenesis in the oral cavity has yet to be well established, but its new classification of (AJCC) could give us more standardization in treatments.

## Case Report

Elderly male patient with a history of exposure to chemical textile agents, smoking, and chronic trauma to the oral mucosa presented a not painful, dark purple-brownish, 3cm x 4cm growth in the left lateral edge of the tongue with spontaneous bleeding. An incisional biopsy was performed, which resulted in the Oral melanoma diagnosis. After other tomographic studies were asked, the patient underwent a hemiglossectomy and cervical dissection. Also, the patient received six cycles of radiotherapy and chemotherapy with Temozolomide + cisplatin. To date, no recurrence has been found.

## Conclusions

The OMM represents a real challenge in our environment due to its different differential diagnoses, such as smokers’ melanosis or amalgam tattoos, which should always be considered in our environment due to its high frequency of presentation. We believe that the best treatment is prevention, and we encourage such important training of a health professional in this area of oral medicine.

# Abstract: Odontogenic myxoma of rare location in the maxilla. Report of a clinical case

**Authors:** R.C. Sánchez Villalba, M. Soler Chaparro, J.M. Gamarra Insfran

## Introduction

Odontogenic myxoma is a rare benign neoplasm of mesenchymal origin originating from the dental organ, locally invasive but not metastatic. There is no predilection for sex. Its most frequent location is the mandible, and it rarely affects the maxilla. It is commonly diagnosed between the second and third decades of life.

## Case Report

This work aims to report a clinical case of odontogenic myxoma in the maxilla and a literature review. A 23-year-old male patient, single, a businessman, came to the Department of Oral Pathology of the Faculty of Dentistry of the National University of Asuncion for presenting a deformity in the facial region on the right side, present since childhood with slow growth and no painful symptoms. An incisional biopsy was performed, and the sample was sent for histopathological examination. The result was a mesenchymal odontogenic tumor compatible with odontogenic myxoma. The treatment was a maxillectomy with safety margins, and the anatomopathological study of the entire excised tumor confirmed the diagnosis of odontogenic myxofibroma with a maximum diameter of 2.5 cm.

## Conclusions

Odontogenic myxoma could be considered as differential diagnosis of radiolucent lesions in maxilla in young patients. The clinical, radiographic, and histopathological correlation is important for the correct diagnosis and treatment of this tumor.

# Abstract: Glandular odontogenic cyst with prominent mucous cells

**Authors:** G.L. Santos, V. Pollo, D.T. Oliveira

## Introduction

The glandular odontogenic cyst is a rare developmental cyst of odontogenic origin that presents glandular differentiation and high recurrence potential. The objective of this case report is to present the clinicopathological characteristics of a glandular odontogenic cyst.

## Case Report

A 74-year-old female patient came to the office for a dental evaluation. On intraoral examination, there were no significant clinical changes. A cone beam computed tomography was requested for planning dental implants, where a hypodense multilocular lesion was observed in the anterior region of the mandible, extending from the alveolar process to the base of the mandible with a slight expansion of the cortical bone. An incisional biopsy was performed, and the material was sent for histopathological analysis. Microscopically, a virtual cystic cavity lined by stratified squamous epithelium was observed, sometimes with a cuboidal aspect of variable thickness and focal plate-like thickening. It was also noted in the epithelium several prominent mucous cells that were positive for PAS, intraepithelial cysts, superficial cells with a “hobnail” pattern, and some cells with clear cytoplasm in the basal layer. In the fibrous capsule, foci of hemosiderosis and absence of inflammation were observed. The final diagnosis was a glandular odontogenic cyst, and the dentist was instructed on the importance of patient follow-up due to the high recurrence rate of the lesion.

## Conclusions

As glandular odontogenic cyst is a rare lesion with unspecific clinical and radiographic characteristics, histopathological analysis becomes essential for correct diagnosis.

# Abstract: Lipoma in the maxillofacial region: Case report

**Authors:** C. Schipper Schwartz, G. Gaffrée Braz, L Moreira de Lucena, E. Almeida Borges de Araújo, R. Ramoa Pires, A.M. Menezes Aguiar Miranda

## Introduction

Lipoma is a benign tumor composed of adipose tissue cells (adipocytes) accumulating inside a fibrous capsule under the skin in the subcutaneous tissue. It is uncommon in the maxillofacial region, with the face and neck region being affected in only 1 to 4% of all cases. Lipomas are mobile nodules on palpation and have a rounded appearance, regular edges, firm and elastic consistency, slow growth, and are almost always painless.

## Case Report

The present work is the clinical case of a male patient, 58 years old, leucoderma, who attended the oral and maxillofacial surgery outpatient clinic of the Lourenço Jorge Municipal Hospital with a report of an asymptomatic increase in volume in the submandibular region on the left side with an evolution of 7 months. On clinical examination, there was a palpable swelling with a soft and mobile consistency. The tomographic examination revealed a hypodense image in the left submandibular region in the subcutaneous plane, measuring 34 mm in its greatest diameter. The patient underwent surgical excision of the lesion through the Risdon access. At trans-surgical, the appearance of the lesion resembled fatty tissue, being sent to histopathology, confirming the diagnosis of Lipoma.

## Conclusions

Lipomas may be present; when this occurs, they are easy to treat and have a favorable prognosis.

# Abstract: Secondary syphilis: Clinical case report

**Authors:** C. Schipper Schwartz, R. Gomes dos Santos, M. Da Silva Monteiro, E. Almeida Borges de Araújo, R. Ramoa Pires, A.M. Menezes Aguiar Miranda

## Introduction

Syphilis is a chronic bacterial infection caused by Treponema pallidum, and its main transmission routes are sexual contact and from mother to fetus. Although the genital regions are the most affected, the oral cavity can manifest signs and symptoms and simulate potentially malignant lesions.

## Case Report

This paper reports the case of a 49-year-old female, leukoderma, with a history of the appearance of “white spots” in the oral cavity with an evolution of 2 months. During the anamnesis, the patient reported having an active sexual life and previous vitamin B12 deficiency, confirmed by laboratory tests. After its supplementation, the values normalized. On clinical examination, the patient presented white plaques with an erythematous halo around them involving the apex and lateral edges of the tongue and the upper and lower lips, similar to the mucous plaques’ characteristic of secondary syphilis. After the incisional biopsy, clinical and histopathological analysis of the patient’s lesions was performed, as well as laboratory tests (VDRL), confirming the diagnosis of syphilis.

## Conclusions

Syphilis is a sexually transmitted disease in most cases. Thus, knowing the main oral manifestations of syphilis is essential to intervene in less advanced stages since it is an infection that can spread rapidly and cause death if left untreated.

# Abstract: Lingual Schwannoma in a 23-year-old man

**Authors:** C.L. Soto Godoy, M.B. Recalde Ruiz Diaz, M.E. Ayala Pereira, A.D. Rodriguez Ayala

## Introduction

 Schwannoma is a benign tumor of Schwann cells, encapsulated and well delimited; the tongue is the most commonly affected area. It represents 1% of oral cavity tumors.

## Case Report

Case report of a 23-year-old man taken care of at a Dental School’s public clinic, referred from a dentist’s private practice. The patient reported painless swelling of the tongue within 18 months of evolution. He had no difficulty chewing or speaking. The clinical examination showed a submucosal lesion of hard consistency circumscribed on the edge of the tongue on the left side, approximately 2 cm in diameter, painless and immobile, and a yellowish-white exophytic lesion in the mucosal area. Adjacent to the lesion was a carious process with loss of dental structure of the lower left first molar. As for the extraoral examination, there were no palpable or painful lymph nodes. A complementary laboratory examination was requested, showing all values within the normal ranks. The excisional biopsy was performed under local anesthesia without complications due to the size and location of the lesion. The histopathologic diagnosis was Schwannoma.

## Conclusions

This fortuitous finding in daily clinical practice was due to a meticulous and exhaustive clinical examination of the professional who referred the patient to the Institution due to the clinical characteristics of the lesion and compliance with the WHO protocol for Potentially Malignant Oral Disorders.

# Abstract: Osteolipoma: a rare tumor in the oral cavity

**Authors:** R. Vera Villar, D.A. Escobar Arevalo, S. Sacsaquispe Contreras

## Introduction

Osteolipoma is a benign neoplasm of soft tissues, with only 1 to 4% occurring in the oral cavity. This rare histological variant presents bone formation, and limited knowledge about this tumor etiopathogenesis is available.

## Case Report

A 46-year-old female patient presents a painless mass in the lower genian region, with 12 months of evolution. Intraoral clinical examination, a tumor with defined edges, stony and painless consistency with an approximate size of 3x1.5x1 cm, located in the bottom mucosa of the sulcus on the left side, without adenopathies, was observed. In the panoramic radiography, a mixed image of a rounded shape with an irregular trabecular pattern is observed. The clinical differential diagnosis included osteoma, bone choristoma, and pleomorphic adenoma with ossification. Under local anesthesia, the tumor was completely removed, and it was attached to the periosteum of the adjacent mandibular bone. On microscopic examination with HE stain, a fibrous capsule was observed in the central area, a proliferation of mature adipose cells, surrounded by trabeculae of vital mature bone, leading to the diagnosis of Osteolipoma. Controls were carried out, and the evolution is favorable to date.

## Conclusions

Osteolipoma is an infrequent entity; it should be considered when the patient presents a hard peripheral lesion, radiographically a mixed image, and histologically adipose tissue surrounded by bone trabeculae. Complete surgical excision is the recommended treatment, and its recurrence is infrequent.

# Abstract: Mucocutaneous ulcer EBV positive: a clinical manifestation of the lymphoproliferative process associated with immunosuppression in the management of Rheumatoid Arthritis. A case report

**Authors:** G.C. Vianna Camolesi, A. Pérez Jardon, P. Gándara Vila, A. García García, M. Pérez Sayans, B. Blanco Carrión

## Introduction

Rheumatoid arthritis (RA) is an autoimmune and inflammatory disease, drugs such as corticosteroids and immunosuppressants are used for this treatment. The primary immunosuppressant administered is methotrexate (MTX), which has been previously described as an iatrogenic cause of lymphoproliferative disorders associated with immunodeficiency, among them lymphoproliferative processes of B cells of the mucocutaneous ulcer type derived from immunosuppression. Histologically they resemble lymphoma and are frequently associated with Epstein-Barr virus (EBV) infection. This entity is usually described in the oral cavity and affects patients with immunosuppression (drugs, primary immunodeficiencies, HIV) and/or advanced age.

## Case Report

A 75-year-old woman with a history of rheumatoid arthritis treated with methotrexate (MTX), among other drugs. She presented with severe pain that made it impossible to use her lower dentures. On clinical inspection, a medium-sized ulcer was noted on the floor of the left mouth, well-defined, of soft consistency, and painful on palpation, with an evolution time of approximately five months. An incisional biopsy of the ulcer was performed to determine whether it was related to the use of MTX. The anatomopathological diagnosis, “Lymphoproliferative process compatible with EBVpositive mucocutaneous ulcer,” has made a correct differential diagnosis with a possible lymphoma using specific stains. Given the context, it was decided, together with the rheumatologist, to suspend treatment with the MTX with the intention of spontaneous remission of the lesion.

## Conclusions

These lesions usually regress spontaneously after medication discontinuation and immune recovery. Clinicalpathological correlation is essential to establish the correct diagnosis.
